# Soil pH and Organic Carbon Properties Drive Soil Bacterial Communities in Surface and Deep Layers Along an Elevational Gradient

**DOI:** 10.3389/fmicb.2021.646124

**Published:** 2021-07-30

**Authors:** Qiuxiang Tian, Ying Jiang, Yanan Tang, Yu Wu, Zhiyao Tang, Feng Liu

**Affiliations:** ^1^CAS Key Laboratory of Aquatic Botany and Watershed Ecology, Wuhan Botanical Garden, Chinese Academy of Sciences, Wuhan, China; ^2^Center of Plant Ecology, Core Botanical Gardens, Chinese Academy of Sciences, Wuhan, China; ^3^University of Chinese Academy of Sciences, Beijing, China; ^4^Key Laboratory for Earth Surface Processes, Department of Ecology, College of Urban and Environmental Sciences, Peking University, Beijing, China

**Keywords:** soil bacterial community, soil layer, soil carbon availability, chemical structure, elevational gradient

## Abstract

Elevational gradients strongly affect the spatial distribution and structure of soil bacterial communities. However, our understanding of the effects and determining factors is still limited, especially in the deep soil layer. Here, we investigated the diversity and composition of soil bacterial communities in different soil layers along a 1,500-m elevational gradient in the Taibai Mountain. The variables associated with climate conditions, plant communities, and soil properties were analyzed to assess their contributions to the variations in bacterial communities. Soil bacterial richness and α-diversity showed a hump-shaped trend with elevation in both surface and deep layers. In the surface layer, pH was the main factor driving the elevational pattern in bacterial diversity, while in the deep layer, pH and soil carbon (C) availability were the two main predictors. Bacterial community composition differed significantly along the elevational gradient in all soil layers. In the surface layer, Acidobacteria, Delta-proteobacteria, and Planctomycetes were significantly more abundant in the lower elevation sites than in the higher elevation sites; and Gemmatimonadetes, Chloroflexi, and Beta-proteobacteria were more abundant in the higher elevation sites. In the deep layer, AD3 was most abundant in the highest elevation site. The elevational pattern of community composition co-varied with mean annual temperature, mean annual precipitation, diversity and basal area of trees, pH, soil C availability, and soil C fractions. Statistical results showed that pH was the main driver of the elevational pattern of the bacterial community composition in the surface soil layer, while soil C fractions contributed more to the variance of the bacterial composition in the deep soil layer. These results indicated that changes in soil bacterial communities along the elevational gradient were driven by soil properties in both surface and deep soil layers, which are critical for predicting ecosystem functions under future climate change scenarios.

## Introduction

Soil microbial communities play critical roles in soil biogeochemical and nutrient cycling processes (Schimel and Schaeffer, 2012; Wagg et al., 2014). Shifts in soil microbial communities may lead to significant changes in carbon (C) and nutrient cycling in plant–soil systems. Along an elevational gradient, biotic and abiotic characteristics change dramatically over a short geographic distance (Körner, 2007; Ren C. et al., 2018b). How forest soil bacterial communities respond to environmental changes along the elevational gradient is critical for predicting future ecosystem functions and climate feedbacks (Bryant et al., 2008; Philippot et al., 2010).

Patterns of soil bacterial community distribution along elevational gradients have been well documented in many studies (Bryant et al., 2008; Yao et al., 2017; Ren C. et al., 2018b). However, the conclusions of these studies are rather contradictory: some studies reported a decreasing diversity with elevation (Bryant et al., 2008; Singh et al., 2014), some studies reported the highest diversity at medium elevation (Meng et al., 2013; Ren C. et al., 2018b), and other studies reported no obvious trends with elevation (Bardhan et al., 2012; Yasir et al., 2015). Furthermore, a wide range of biotic and abiotic factors, such as soil pH, soil organic carbon (SOC) content, soil nutrient, plant traits, and temperature, have been shown to influence bacterial diversity and composition along elevational gradients (Liu et al., 2016; Yao et al., 2017; Ren C. et al., 2018b). These inconsistent trends and controlling factors of bacterial diversity and composition might be caused by different study sites and elevation ranges, which affect the variances in environmental characteristics among sites. Broader variances in climatic conditions, and plant and soil properties will be more effective to reveal the elevational pattern of bacterial community and facilitate the evaluation of the relative importance of these factors.

Soil organic carbon availability and quality can regulate the diversity and composition of soil microbial communities (Ding et al., 2015; Delgado-Baquerizo et al., 2016; Tian et al., 2018; Shao et al., 2019). SOC-rich soils usually have higher bacterial diversity than SOC-poor soils. Moreover, different soil bacterial groups have different life strategies. Some groups of bacterial communities (copiotrophic taxa) are known to dominate in soils where SOC is more abundant and decomposable, while some other groups (oligotrophic taxa) usually grow in soils with lower C content, lower labile C pool, and lower C mineralization rates (Fierer et al., 2007; Yao et al., 2017). Thus, SOC content and mineralization rate, which represent soil C availability, are important determinants of soil bacterial diversity and composition (Ding et al., 2015; Yao et al., 2017; Tian et al., 2018). Besides, the chemical components of SOC (known as soil labile or recalcitrant C fractions) are also demonstrated to affect soil bacterial communities (Davinic et al., 2012; Ng et al., 2014; Li et al., 2018; Deng et al., 2019), since individual bacterial taxa exhibit different resource preferences (Paterson et al., 2008; Di Lonardo et al., 2017; Ivanova et al., 2018). For example, Acidobacteria, Delta-proteobacteria, and Gemmatimonadetes can degrade more recalcitrant substrates, while Actinobacteria and Beta-proteobacteria can utilize fresh and labile substrate (Pascault et al., 2013; Chen et al., 2015). Dramatic changes in plant communities and soil physico-chemical properties along elevational gradients are expected to cause strong variations in soil C availability and soil C fractions (Djukic et al., 2010; Du et al., 2014). Determining their effects on soil bacterial diversity and composition along elevational gradients are important to better understand the biogeography of bacterial community and their ecological functions.

Most studies on bacterial communities along an elevational gradient focused solely on the surface layer (0–20 cm), where soil microorganisms were most abundant and active. However, microbial communities in deeper soil layers also play important roles in regulating biogeochemical processes associated with nutrient cycling, soil formation, and contaminant degradation (Fierer et al., 2003; Eilers et al., 2012; Li et al., 2014). Due to the large variations in soil properties and SOC content across soil layers, recent studies have shown that bacterial diversity and composition differed significantly among soil layers (Chu et al., 2016; Nacke et al., 2016; de Araujo Pereira et al., 2017; Du et al., 2017). However, little is known about whether the environmental changes along elevational gradients affect soil bacterial communities in deeper soil layers.

The purpose of this study was (1) to explore the spatial patterns of the diversity and composition of soil bacterial communities along an elevational gradient across soil layers and (2) to reveal their determining factors, especially the factors associated with soil C availability and C fractions. We hypothesized that (1) soil bacterial α-diversities would show hump-shaped trends along the elevational gradient for both surface and deep layers due to the highest soil pH in the medium elevation and (2) soil bacterial composition would vary significantly along the elevational gradient in both surface and deep layers, but the driving factors might differ across soil layers. To test these hypotheses, we carried out a study in the Taibai Mountain in the Qinling Range of central China. We used 16S rRNA gene sequencing to reveal changes in the soil bacterial diversity and composition. The variables associated with climate, vegetation, soil physico-chemical properties, soil C availability, and C fractions were analyzed to assess their effects on the variations in the bacterial communities along the elevational gradient.

## Materials and Methods

### Site Description and Soil Sampling

The study was conducted in the Taibai Mountain (107°19′–107°58′E and 33°45′–34°10′N), which is the peak of Qinling Mountains in central China. The Qinling Mountains vary in elevation from 470 to 3,760 m above sea level, and they are the climate demarcation line between the northern and southern regions of China as well as the watershed between the Yellow and Yangtze Rivers. The northern slope of the Taibai Mountain falls into five climate zones: warm temperate, temperate, cold temperate, cold, and alpine cold. The average annual temperature varies from 11.0°C (1,250 m a.s.l.) to 1.1°C (3,250 m a.s.l.), and the mean annual precipitation (MAP) ranges from 600 to 1,000 mm, with the highest precipitation in the middle elevation of 1,850–2,400 m a.s.l. These diverse environmental factors cause a variety of forest types along the elevational gradient, including *Quercus aliena* var. *acuteserrata* forest (1,200–1,800 m a.s.l.), *Quercus liaotungensis* forest (1,800–2,300 m a.s.l.), *Betula albo-sinensis* forest (2,300–2,600 m a.s.l.), *Abies fargesii* forest (2,600–3,000 m a.s.l.), and *Larix chinensis* forest (3,000–3,400 m a.s.l.).

We sampled soils at five sites on the northern slope of the Taibai Mountain with average elevations of 1,649, 2,139, 2,443, 2,872, and 3,145 m a.s.l. (Supplementary Figure 1). The five sites were chosen to cover the five forest types. The site of *Q. aliena* var. *acuteserrata* forest was about 0.8, 14.4, 13.5, and 13.1 km away from sites of *Q. liaotungensis* forest, *B. albo-sinensis* forest, *A. fargesii* forest, and *L. chinensis* forest, respectively (Supplementary Table 1). For each site, three plots (20 × 50 or 30 × 33 m depending on site conditions) were established for vegetation investigation and soil sampling. The three plots are at least 50 m away from each other. At each plot, all trees with a diameter at breast height (DBH) ≥ 5 cm were identified at species level, and the DBH and height of each tree were measured. Tree density, sum of breast-height basal area, and Shannon’s index of the tree species were calculated. Mean annual temperature (MAT) was calculated according to an empirical formula (the northern slope: MAT = −0.00495 × elevation + 17.1875) provided by literature (Tang and Fang, 2006). MAP was calculated following the relationship between MAP and elevation in mountain regions (Fu, 1982). Detailed information regarding climate conditions, vegetation properties, and soil type of each forest is given in Table 1.

**TABLE 1:** The properties of the sampling sites along the northern slope of Taibai Mountain.

Elevation (m a.s.l.)	Forest types	MAT (°)	MAP (mm)	Tree Shannon’s index	Tree Simpson’s index	Tree basal area (cm^2^ m^–2^)	Tree density (trees ha^–1^)	Soil types
1,571–1,721	*Quercus aliena* var. *acuteserrata* forest	9.0 ± 0.3e	854 ± 9b	0.80 ± 0.37b	0.36 ± 0.20b	37.0 ± 5.4c	1,337 ± 249ab	Hapli-Udic Argosols
2,075–2,193	*Quercus liaotungensis* forest	6.6 ± 0.3d	890 ± 2c	1.16 ± 0.31b	0.55 ± 0.14b	33.3 ± 3.9bc	1,647 ± 411ab	Hapli-Udic Argosols
2,423–2,458	*Betula albo-sinensis* forest	5.1 ± 0.1c	890 ± 0c	1.31 ± 0.11b	0.65 ± 0.06b	13.4 ± 4.3a	556 ± 315a	Hapli-Udic Argosols
2,819–2,945	*Abies fargesii* forest	3.0 ± 0.3b	863 ± 7b	1.05 ± 0.28b	0.62 ± 0.10b	23.9 ± 5.9ab	1,920 ± 730b	Acid-Udic Cambisols
3,120–3,175	*Larix chinensis* forest	1.6 ± 0.1a	828 ± 4a	0a	0a	20.0 ± 6.2a	853 ± 311ab	Acid-Udic Cambisols

*Results are means ± standard deviations for *n* = 3. Different lowercase letters indicate a significant difference among sites (*p* < 0.05). Soil was classified according to the Chinese genetic soil classification. MAT, mean annual temperature; MAP, mean annual precipitation; Tree basal area, the sum of breast-height basal areas of trees.*

One soil profile at each plot was dug in October 2016. Surface organic material was carefully removed. For each profile, soil samples were collected according to their mineral horizons. In *Q. aliena* forest and *Q. liaotungensis* forest, four soil layers were collected (A, AB, B1, and B2). In *B. albo-sinensis* forest, *A. fargesii* forest, and *L. chinensis* forest, three soil layers were collected (A, AB, and B). Overall, 51 soil samples were collected (2 forest sites × 3 plots × 4 layers + 3 forest sites × 3 plots × 3 layers). For each sample, about 2 kg of fresh soil was collected. All soil samples were then immediately brought to the laboratory and passed through a 2-mm sieve. Roots and visible residues were picked out manually, and the roots were thoroughly washed with deionized water and dried at 65°C for 48 h to calculate root biomass in mg g^–1^ dry soil. Part of the soils was stored at −80°C for genomic DNA extraction, PCR, and sequencing. Part of the soils was stored at 4°C for dissolved organic C and nitrogen analyses within 2 days. The other part of the soils was air-dried for further physico-chemical analysis.

### Soil Properties

Soil organic carbon and total nitrogen (TN) were measured using an elemental analyzer (Flash 2000, Thermo Fisher Scientific Inc., Waltham, MA, United States). Before C and nitrogen analyses, soil samples were tested for the presence of carbonate, and no carbonate was found. Concentrations of soil inorganic nitrogen (SIN, NH_4_^+^–N + NO_3_^–^–N) were extracted with 2 mol L^–1^ of KCl and then measured using a discrete autoanalyzer (EasyChem, Systea Scientific Inc., Rome, Italy).

Soil organic carbon was further fractionated into different C pools with physical and chemical methods. First, SOC was fractionated into particulate organic C (POC) using size fractionation (>53 μm). Second, a two-step acid hydrolysis procedure with H_2_SO_4_ was used to separate SOC into labile and recalcitrant C pools, which are featured by different chemical compositions (Rovira and Vallejo, 2002). Briefly, soil samples were first hydrolyzed with 2.5 mol L^–1^ of H_2_SO_4_ at 105°C for 30 min. The organic C in this hydrolyzate was taken as labile pool I (LPI). The remaining residue was further hydrolyzed with 13 mol L^–1^ of H_2_SO_4_ and shaken overnight at room temperature. The concentration of the acid was then brought down to 1 mol L^–1^ by dilution with de-ionized water, and the sample was hydrolyzed for 3 h at 105°C with occasional shaking. The organic C in this second hydrolyzate was taken as labile pool II (LPII), and C in the residue was measured as recalcitrant pool (RP). The relative abundances of LPI, LPII, and RP were calculated as the ratio of SOC in each pool to the total SOC. Among the three fractions, LPI; is known to predominantly contain carbohydrates such as polysaccharides, hemicellulose, and soluble sugars; LPII is largely cellulose; and RP is mainly composed of aromatic C fractions (Oades et al., 1970). Total organic C (TOC) contents in the hydrolyzates were measured using a TOC Analyzer (Vario TOC, Elementar, Langenselbold, Germany). Organic C contents in the solid residues were then determined with an elemental analyzer (Thermo Fisher Flash 2000, United States).

Soil pH was measured with a calomel electrode on a paste of 1:2.5 (weight:volume) of air-dried soil and deionized water. Soil texture was determined with a laser particle size analyzer (Mastersizer 3000, Malvern Panalytical, Malvern, United Kingdom). The amount of poorly crystalline Fe and Al oxides was determined by extraction with acid ammonium oxalate at pH 3 in the dark (oxalate extraction, Fe_*o*_ and Al_*o*_). Fe and Al concentrations in the extractions were quantified by plasma atomic emission spectrometry (Optima 8000DV, PerkinElmer, Waltham, MA, United States). The mole ratio of poorly crystallized Fe and Al to SOC (M_*o*_:SOC) was applied to represent soil C availability, with a high M_*o*_:SOC ratio representing low C availability (Gentsch et al., 2018; Chen et al., 2019).

### Soil Bacterial Communities

Soil genomic DNA was extracted from 0.5 g of fresh soil samples using a MoBio PowerSoil DNA Isolation extraction kit (MoBio Laboratories, Carlsbad, CA, United States). The extracted DNA samples were diluted to 10 ng μl^–1^ for further use. The V4–V5 region of prokaryotic 16S rRNA gene primer pairs 515F (5′-GTGYCAGCMGCCGCGGTA-3′)/909R (5′-CCCCGYCAATTCMTTTRAGT-3′) with barcodes were selected for PCR amplification. The PCR program for 16S rRNA gene included initial denaturation at 94°C for 3 min, followed by 30 cycles of 94°C for 40 s, 56°C for 60 s, and 72°C for 60 s, and a final extension at 72°C for 10 min. Three replicate PCR products for each sample were combined and loaded in 1.0% agarose gel electrophoresis. The correct bands were excised and purified with gel extraction kits (Sangon Biotech, Shanghai, China; Cat#SK8135). Sequencing was conducted using an Illumina (San Diego, CA, United States) MiSeq platform for sequencing (Reagent Kit V2) on the Environmental Genome Platform of Chengdu Institute of Biology, CAS. In total, 1,180,008 raw sequences were generated, and the average sequencing depth ranged from 10,161 to 38,950 reads per sample.

Sequence data were spliced with FLASH and sorted by barcodes (Magoč and Salzberg, 2011). Barcoded sequences with length < 200 bp and average Phred quality scores < 30 were culled. Chimeras were removed using UCHIME algorithm implemented in USEARCH (version 8) with database SILVA v128 (version SSU^1^) for 16S rRNA gene. QIIME V1.9.0^2^ was used to analyze sequencing data after splicing and quality control. Sequences were classified taxonomically using the Ribosomal Database Project (version 2.2^3^) database. Clustering of operational taxonomic units (OTUs) at 97% similarity was performed with the open-reference OTU picking method. Sequences classified as mitochondria or chloroplast were removed. Sequences for each sample were subsampled to the same sequence depth (7,908 reads) to ensure comparability among samples. Representative sequences were aligned by using PyNAST software in QIIME (Caporaso et al., 2010). Then gaps were filtered with the default parameters in script filter_alignment.py. Phylogenetic maximum likelihood-approximation tree was reconstructed using the generalized time-reversible model in FastTree (Price et al., 2009). The raw sequencing data were archived at the National Center for Biotechnology Information^4^, in the Sequence Read Archive (SRA) database (accession number PRJNA574939).

### Statistical Analysis

The OTU Table generated in QIIME was used to quantify the OTU richness (observed OTUs), α-diversity (Shannon’s index and Faith’s phylogenetic diversity) and β-diversity (weighted UniFrac pairwise distances between samples). Non-metric multidimensional scaling (NMDS) ordinations were used to visualize the weighted UniFrac pairwise distances matrices among elevations and soil layers using the “vegan” (Oksanen et al., 2019) and “ggplot2” packages (Wickham, 2016) in R^5^.

The differences in MAT, MAP, basal area of trees, tree density, Shannon’s index, and Simpson’s index of trees, and soil properties in each layer among the five sites were compared by one-way analyses of variance (ANOVAs) with Tukey’s honestly significant difference (HSD) as *post hoc* comparisons. When considering the effects of elevation and soil layer on soil properties, bacterial α-diversity, and the relative abundance of bacterial dominant phyla, linear mixed-effects models were employed in R “lme4” package (Bates et al., 2015) with sampling site (i.e., five forest sites) and soil layer as fixed-effect factors and the sampling plot as a random-effect factor to account for non-independence of samples collected in different soil layers. Permutational multivariate analysis of variance (PerMANOVA) was employed in Past 3 software to determine the influence of elevation and soil layer on bacterial β-diversity.

We further analyzed the elevational patterns of soil bacterial communities in different soil layers. Soils from mineral A, AB, and B layers were considered as surface soil, subsurface soil, and deep soil, respectively. In *Q. aliena* forest and *Q. liaotungensis* forest, the B layer was collected as two sublayers (B1 and B2 layers). Since soil physico-chemical properties and bacterial community structure were similar between B1 and B2 layers in the two forests; B2 soils were used to represent deep layer soils. In this way, a total of 45 soil samples (5 sites × 3 plots × 3 layers) were used for later statistical analyses. In each layer, linear and non-linear (polynomial) regressions were used to analyze the relationships between bacterial α-diversity and elevation. Spearman’s and partial Spearman’s correlations were used to analyze the correlations between bacterial α-diversity, relative abundance of bacterial dominant taxa, and the measured environmental variables in R “psych” (Revelle, 2021) and “ppcor” (Kim, 2015) packages. Mantel and partial Mantel tests at 999 permutations were further used to determine the impact of the measured environmental variables (Euclidean distance) on bacterial β-diversity in PASSaGE 2 software. *p*-Values of these correlation analyses were adjusted for multiple testing according to the Benjamini and Hochberg (1995) method. Since geographic distances among sites are highly correlated with elevation (or MAT) (*p* < 0.001), the effect of geographic distance was not considered and discussed.

To assess the relative contributions of environmental variables to the variance of soil bacterial composition along the elevational gradient, a variation partitioning method based on constrained and partial canonical ordination techniques was adopted using the “vegan” package (Oksanen et al., 2019) in R. Three groups of explanatory factors (climate conditions, vegetation properties, and soil properties) were summarized for variation partitioning to assess their contributions to the variance of soil bacterial community. Three groups of soil properties (pH, soil C availability, and soil C fractions) were further summarized for variation partitioning to assess the contributions of different soil properties to the variance of soil bacterial community.

## Results

### Vegetation and Soil Properties Along the Elevational Gradient

The sum of basal area of trees followed a decreasing trend with elevation (*r* = −0.579, *p* = 0.024, Table 1 and Supplementary Table 2). Tree density had no significant correlation with elevation (*r* = −0.239, *p* = 0.390), but *A. fargesii* forest had a significant higher tree density than *B. albo-sinensis* forest. Tree diversity and richness in the highest elevation site (*L. chinensis* forest) were significantly lower than in the other four sites.

Soil physico-chemical properties were significantly affected by elevation, soil layer, or their interaction effects (Table 2). In the surface layer, SOC, TN, C:N ratio POC%, and RP% showed an increasing trend with elevation; and M_*o*_:SOC ratio and LPI% showed an opposite trend (Supplementary Table 2). The soils at the medium elevation site (2,423–2,458 m a.s.l.) had the highest percentage of sand particles. In the subsurface layer, only pH showed an increasing trend with elevation (Supplementary Table 3). In the deep layer, SOC, TN, C:N ratio, and SIN showed an increasing trend with elevation (Supplementary Table 4). In addition, SOC, TN, C:N, M_*o*_:SOC, RP%, and LPI% were correlated with each other in the surface layer (Supplementary Table 2). In the deep layer, SOC, TN, and M_*o*_:SOC were correlated with each other but showed no significant correlations with RP% and LPI% (Supplementary Table 4). Soil pH had no significant correlations with other soil physico-chemical properties in either layer.

**TABLE 2:** Key properties of the soil samples taken from the five forest types along the northern slope of Taibai Mountain.

Elevation (m a.s.l.)	Layer	Depths (cm)	SOC (mg g^–1^ d.w.)	TN (mg g^–1^ d.w.)	C:N	SIN (μg g^–1^ d.w.)	POC (%)	LPI (%)	LPII (%)	RP (%)	M_*o*_:SOC	Clay (%)	Sand (%)	pH
1,571–1,721	A	0–15	18.3 ± 8.1	1.4 ± 0.6	13.3 ± 0.4	2.7 ± 0.5	23.2 ± 12.6	29.3 ± 3.3	11.7 ± 1.7	59.0 ± 5.0	0.10 ± 0.03	13.8 ± 0.8	28.6 ± 4.3	5.1 ± 0.2
	AB	15–30	9.3 ± 7.0	0.8 ± 0.6	11.4 ± 1.2	2.3 ± 0.7	14.2 ± 6.5	30.4 ± 6.6	11.3 ± 0.5	58.3 ± 6.1	0.20 ± 0.11	13.3 ± 2.5	31.0 ± 8.9	5.4 ± 0.3
	B1	30–50	5.6 ± 4.5	0.5 ± 0.4	10.9 ± 0.3	1.4 ± 0.3	9.0 ± 5.2	38.0 ± 12.1	10.5 ± 1.5	51.5 ± 13.4	0.41 ± 0.26	12.4 ± 3.8	33.2 ± 11.7	5.7 ± 0.3
	B2	50–79	3.6 ± 1.7	0.4 ± 0.2	9.7 ± 0.3	0.8 ± 0.1	8.6 ± 3.8	40.8 ± 4.7	10.9 ± 1.5	48.3 ± 5.6	0.51 ± 0.16	14.2 ± 3.0	28.9 ± 6.8	5.6 ± 0.4
2,075–2,193	A	0–15	32.2 ± 0.8	2.6 ± 0.1	12.2 ± 0.1	2.3 ± 0.5	24.5 ± 4.7	23.2 ± 1.2	11.9 ± 1.4	64.9 ± 2.3	0.06 ± 0.01	15.2 ± 2.1	26.5 ± 1.9	6.2 ± 0.4
	AB	15–34	18.9 ± 6.5	1.8 ± 0.5	10.3 ± 1.1	1.8 ± 0.4	14.1 ± 1.1	29.4 ± 2.1	14.3 ± 2.5	56.3 ± 0.8	0.12 ± 0.05	16.0 ± 0.7	27.4 ± 2.5	5.9 ± 0.5
	B1	34–57	13.5 ± 0.8	1.3 ± 0.0	10.2 ± 0.4	1.4 ± 0.4	8.5 ± 2.4	35.1 ± 3.8	14.6 ± 3.6	50.3 ± 6.1	0.16 ± 0.01	16.1 ± 0.6	25.7 ± 0.4	6.1 ± 0.2
	B2	57–95	6.1 ± 3.4	0.6 ± 0.3	10.0 ± 1.2	1.2 ± 0.1	6.7 ± 2.8	42.0 ± 3.1	14.9 ± 2.6	43.1 ± 2.2	0.32 ± 0.11	13.8 ± 2.1	31.5 ± 9.8	6.2 ± 0.3
2,423–2,458	A	0–18	60.4 ± 27.8	4.5 ± 1.7	13.2 ± 1.2	3.3 ± 1.4	27.6 ± 24.4	22.7 ± 3.2	9.8 ± 1.0	67.5 ± 3.4	0.04 ± 0.02	12.3 ± 2.6	40.0 ± 11.8	6.3 ± 0.3
	AB	18–33	27.5 ± 20.4	2.4 ± 1.7	11.4 ± 0.3	2.7 ± 1.0	10.5 ± 4.6	32.8 ± 5.1	12.3 ± 2.0	54.9 ± 7.0	0.10 ± 0.05	11.0 ± 2.8	45.2 ± 11.0	6.4 ± 0.3
	B	33–71	9.0 ± 4.3	0.8 ± 0.4	11.7 ± 1.1	1.3 ± 0.4	6.7 ± 2.5	42.8 ± 4.2	12.9 ± 1.0	44.2 ± 5.0	0.21 ± 0.05	8.5 ± 3.4	51.8 ± 11.1	6.6 ± 0.2
2,819–2,945	A	0–17	78.2 ± 22.4	5.2 ± 1.2	15.0 ± 1.1	2.8 ± 0.7	37.2 ± 10.3	19.4 ± 1.3	13.0 ± 1.2	67.6 ± 2.1	0.04 ± 0.01	17.1 ± 1.1	21.7 ± 2.2	6.0 ± 0.7
	AB	17–34	36.0 ± 13.9	2.9 ± 1.0	12.5 ± 0.8	1.6 ± 0.3	14.3 ± 4.1	30.1 ± 4.4	16.3 ± 2.8	53.6 ± 4.3	0.10 ± 0.03	17.3 ± 0.8	21.3 ± 1.6	6.1 ± 0.7
	B	34–61	13.0 ± 2.6	1.2 ± 0.2	11.1 ± 0.6	1.1 ± 0.2	6.8 ± 1.4	37.3 ± 6.5	14.7 ± 3.4	48.1 ± 3.1	0.23 ± 0.03	16.6 ± 1.9	20.4 ± 3.1	6.4 ± 0.4
3,120–3,175	A	0–17	134.1 ± 49.7	7.6 ± 1.9	17.3 ± 2.4	3.0 ± 1.3	47.4 ± 12.9	20.9 ± 4.4	8.4 ± 1.5	70.7 ± 5.8	0.03 ± 0.01	14.1 ± 0.3	28.3 ± 3.6	5.9 ± 0.3
	AB	17–40	19.5 ± 7.6	1.6 ± 0.7	12.0 ± 1.5	2.6 ± 0.8	4.7 ± 2.9	37.8 ± 4.7	11.4 ± 0.4	50.9 ± 4.8	0.21 ± 0.06	9.8 ± 1.4	45.7 ± 6.5	6.3 ± 0.1
	B	40–72	11.6 ± 2.4	0.9 ± 0.2	12.5 ± 1.0	1.5 ± 0.2	2.9 ± 0.8	51.0 ± 5.7	10.0 ± 0.4	39.0 ± 6.0	0.35 ± 0.10	9.5 ± 0.9	48.6 ± 3.8	6.4 ± 0.1
***p*-value of the LMMs**														
Elevation			**<0.001**	**<0.001**	**<0.001**	0.207	0.728	0.102	**0.003**	0.883	**0.011**	**<0.001**	**<0.001**	**<0.001**
Layer			**<0.001**	**<0.001**	**<0.001**	**<0.001**	**<0.001**	**<0.001**	**<0.001**	**<0.001**	**<0.001**	**<0.001**	**<0.001**	**<0.001**
Elevation × Layer			**<0.001**	**<0.001**	**0.002**	0.344	**<0.001**	**0.002**	**0.008**	**<0.001**	**0.011**	**0.001**	**<0.001**	0.376

*Results are means ± standard deviations for *n* = 3. The bottom half of the table shows the effects of forest type and soil layer on the parameters by using linear mixed models (LMMs). Numbers in bold indicate significant effects with *p* < 0.05. d.w., dry weight of soil; SIN, soil inorganic nitrogen (NH_4_^+^ + NO_3_^–^); POC, particulate soil organic carbon; LPI, labile carbon pool I; LPII, labile carbon pool II; RP, recalcitrant carbon pool; M_*o*_:SOC, mole ratio of poorly crystallized Fe and Al to SOC.*

Soil organic carbon, TN, C:N ratio, clay particle, POC%, and RP% decreased from surface soil to deep soil, while LPI;%, pH, M_*o*_:SOC ratio, and sand particle showed an opposite trend (Table 2).

### Soil Bacterial Community in Relation to Elevation and Soil Layer

**FIGURE 1:**
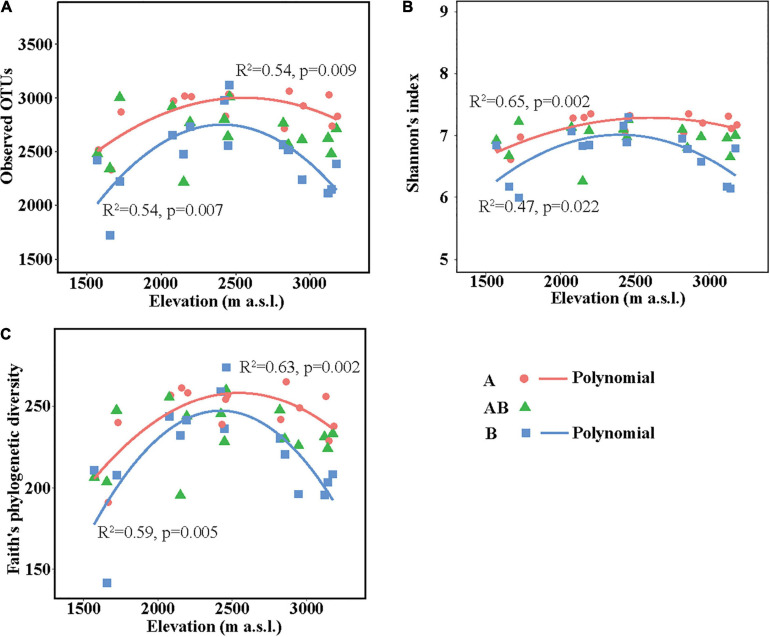
Soil bacterial community richness and α-diversity along the elevational gradient. **(A)** Observed operational taxonomic units (OTUs), **(B)** Shannon’s diversity, and **(C)** Faith’s phylogenetic diversity. Lines in each plot represent the least squares regression fits. OTUs, operational taxonomic units.

The richness (represented by observed OTUs) and α-diversity (represented by Shannon’s diversity and Faith’s phylogenetic diversity) of soil bacterial communities differed among elevation sites and soil layers (Supplementary Table 5). In the surface layer, the three indices in the lowest elevation site (1,571–1,721 m a.s.l.) were lower than those in the other elevation sites. In the subsurface layer, the three indices were all similar among the five elevation sites. In the deep layer, soils in the medium elevation site (2,423–2,458 m a.s.l.) had the highest bacterial richness and α-diversity. Overall, the three indices showed no significant linear correlations with elevation in each layer but showed significant hump-shaped trends with elevation in the surface and deep layers (Figure 1).

**FIGURE 2:**
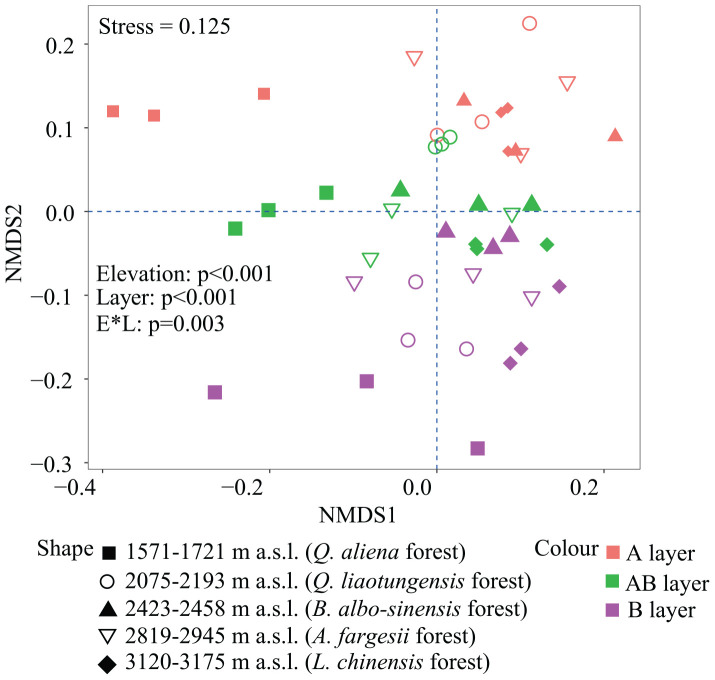
Non-metric multidimensional scaling (NMDS) of soil bacterial community based on weighted UniFrac distances derived from OTU composition. OTUs, operational taxonomic units.

Non-metric multidimensional scaling was conducted to reflect bacterial β-diversity among experimental groups (Figure 2 and Supplementary Figure 2). Bacterial community composition differed significantly across the five elevation sites and soil layers (PerMANOVA, elevation, *p* < 0.001; layer, *p* < 0.001). Surface soils showed a larger variation in bacterial community composition than the deeper soils. Elevation (i.e., MAT) was significantly correlated with soil bacterial β-diversity in each soil layer (Table 3).

**TABLE 3:** Mantel and partial Mantel tests of soil bacterial β-diversity with environmental parameters.

Parameters	Surface layer		Subsurface layer		Deep layer	
	Mantel	Partial Mantel	Mantel	Partial Mantel	Mantel	Partial Mantel
MAT	0.580*	0.393*	0.648**	0.433**	0.595*	0.524*
MAP	0.016	−0.047	**0.272**	0.239	0.212	−0.167
Div	−0.100	−0.01	0.136	−0.064	0.344*	0.346*
TD	−0.128	0.063	−0.149	−0.084	−0.313	**−0.311**
BA	0.346*	0.055	**0.273**	0.031	**0.282**	−0.146
SOC	0.115	−0.144	0.120	−0.232	0.291*	0.074
M_*o*_:SOC	0.695*	0.424	0.260	0.024	0.591*	**0.396**
C:N	−0.108	0.057	0.067	0.063	0.193	−0.041
TN	0.298	0.026	0.179	0.263	0.204	−0.108
SIN	−0.096	0.069	−0.223	−0.236	0.157	**−0.317**
POC%	−0.020	−0.123	0.072	0.224	0.454*	0.163
RP%	0.484*	0.153	0.006	−0.018	0.028	0.263
LPI%	0.577*	0.081	0.018	−0.006	−0.055	**−0.317**
LPII%	−0.047	0.07	−0.041	0.172	0.089	0.285
pH	0.630*	0.708*	0.482**	0.592**	0.504*	0.218
Clay	−0.139	−0.071	−0.071	−0.145	0.028	0.238
Sand	−0.109	0.121	−0.084	0.123	−0.025	−0.04

Dissimilarity matrices of environmental characteristics were calculated based on Euclidean distances. β-Diversity of bacterial community was calculated based on weighted UniFrac distances. Numbers in bold indicate significant relationships at *p* < 0.1. *p < 0.05; **p < 0.01. *p*-Values were corrected for multiple testing using the Benjamini and Hochberg method. MAT, mean annual temperature; MAP, mean annual precipitation; Div, Shannon’s index of trees; BA, the sum of breast-height basal areas of trees; TD, tree density; SIN, soil inorganic nitrogen (NH_4_^+^ + NO_3_^–^); POC%, proportion of particulate organic carbon in SOC; LPI%, proportion of labile carbon I in SOC; LPII%, proportion of labile carbon II in SOC; RP% proportion of recalcitrant carbon in SOC; M_*o*_:SOC, mole ratio of poorly crystallized Fe and Al to SOC.

**FIGURE 3:**
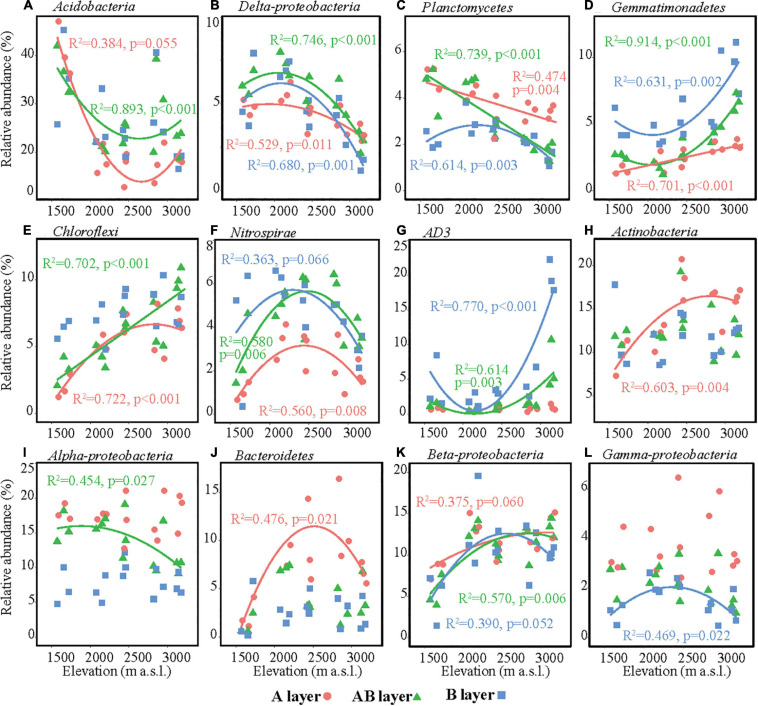
Relative abundances of the dominant bacterial taxa along the elevational gradient. **(A)** Acidobacteria, **(B)** Delta-proteobacteria, **(C)** Planctomycetes, **(D)** Gemmatimonadetes, **(E)** Chloroflexi, **(F)** Nitrospirae, **(G)** AD3, **(H)** Actinobacteria, **(I)** Alpha-proteobacteria, **(J)** Bacteroidetes, **(K)** Beta-proteobacteria, **(L)** Gamma-proteobacteria. Lines in each plot represent the least squares regression fits.

The main bacterial taxa (relative abundance > 3%) were Acidobacteria, Actinobacteria, AD3, Alpha-proteobacteria, Beta-proteobacteria, Bacteroidetes, Chloroflexi, Delta-proteobacteria, Gamma-proteobacteria, Gemmatimonadetes, Nitrospirae, and Planctomycetes (Figure 3). The relative abundances of some main bacterial taxa were significantly affected by elevation, soil layer, and their interaction effect (Supplementary Table 5). In the surface layer, the relative abundances of Acidobacteria and Delta-proteobacteria in the lowest elevation site (1,571–1,721 m a.s.l.) were significantly higher than those in the highest elevation, while the relative abundances of Chloroflexi, Actinobacteria, Bacteroidetes, and Beta-proteobacteria in the lowest elevation site (1,571–1,721 m a.s.l.) were significantly lower than those in the other elevation sites (Figure 3). All these six bacterial taxa showed significant hump-shaped trends with elevation (Figure 3). The relative abundances of Planctomycetes showed a decreasing trend, and Gemmatimonadetes showed an increasing trend with elevation. In the subsurface and deep layers, the differences in the relative abundances of these taxa among elevation sites decreased or even disappeared, except for AD3. The relative abundance of AD3 in the highest elevation site (3,120–3,175 m a.s.l.) was higher than that in the other sites in the subsurface and deep layers. In the deep layer, most of the bacterial taxa showed significant hump-shaped trends with increased elevation (Figure 3).

At the class level, Chloracidobacteria, DA052, Thermoleophilia, Acidobacteriia, Nitrospirae, Saprospirae, Actinobacteria, iii1-8, Ellin6529, ABS-6, Planctomycetia, Solibacteres, Acidobacteria-6, and Gemm-1 were the main taxa (relative abundance > 2%, Supplementary Figure 3). The bacterial taxa at the class level have similar elevational patterns with their corresponding phyla except for Chloracidobacteria, iii1-8, Acidobacteria-6, and Actinobacteria, which showed contrasting trends (Supplementary Figure 3).

### Soil Bacterial Diversity in Relation to Environmental Variables

In the surface layer, we only found a slight correlation between pH and Shannon’s index of bacterial community (Table 4). In the subsurface layer, bacterial α-diversities were only slightly correlated with M_*o*_:SOC, while deep soil bacterial α-diversities were significantly correlated with MAP, Shannon’s index of trees, LPII%, M_*o*_:SOC, and pH. Partial correlation analysis was performed for the five variables in the deep layer, and pH and LPII% were identified as the significant variables (Supplementary Table 6).

**TABLE 4:** Spearman’s rank correlations between soil bacterial richness and diversity (Shannon’s diversity and phylogenetic diversity) and environmental parameters.

Parameters	Surface layer			Subsurface layer			Deep layer		
	Observed OTUs	Shannon’s diversity	Faith’s PD	Observed OTUs	Shannon’s diversity	Faith’s PD	Observed OTUs	Shannon’s diversity	Faith’s PD
Elevation	0.275	0.371	0.096	0.014	−0.086	0.093	−0.118	−0.136	−0.196
MAT	−0.275	−0.371	−0.096	−0.014	0.086	−0.093	0.118	0.136	0.196
MAP	0.366	0.413	0.549	0.379	0.345	0.313	0.786**	0.665*	0.801**
Div	0.258	0.315	0.366	0.498	0.538	0.380	0.703*	**0.584**	0.642*
TD	−0.111	−0.061	0.288	−0.061	0.029	0.023	−0.08	−0.175	−0.182
BA	−0.425	−0.386	−0.146	−0.300	−0.218	−0.321	−0.282	−0.304	−0.261
SOC	0.221	0.304	0.004	0.368	0.343	0.425	−0.064	−0.204	−0.171
M_*o*_:SOC	−0.143	−0.175	0.096	**−0.629**	**−0.646**	**−0.571**	−0.639*	−0.439	−0.561
C:N	−0.261	−0.264	−0.511	−0.189	−0.207	−0.146	−0.102	−0.089	−0.104
TN	0.221	0.304	0.004	0.436	0.432	0.486	−0.014	−0.161	−0.136
SIN	0.232	0.282	0.011	0.525	0.550	0.414	0.243	0.189	0.279
POC%	0.032	0.107	−0.179	−0.277	−0.161	−0.324	0.136	0.086	0.129
RP%	0.171	0.250	0.043	0.150	0.189	0.107	−0.100	−0.254	−0.136
LPI%	−0.171	−0.325	−0.179	−0.239	−0.254	−0.243	−0.239	−0.046	−0.175
LPII%	−0.036	0.068	0.314	0.096	0.061	0.182	0.847**	0.744*	0.745*
pH	0.384	**0.563**	0.540	0.439	0.336	0.554	**0.618**	0.636*	0.543
Clay	0.107	0.050	0.286	−0.143	−0.075	−0.061	−0.218	−0.396	−0.311
Sand	0.198	0.080	−0.050	0.296	0.211	0.218	0.254	0.350	0.364

Dissimilarity matrices of the environmental characteristics were calculated based on Euclidean distances. Numbers in bold indicate significant relationships at *p* < 0.1. *p < 0.05; **p < 0.01. *p*-Values were corrected for multiple testing using the Benjamini and Hochberg method. MAT, mean annual temperature; MAP, mean annual precipitation; Div, Shannon’s index of trees; BA, the sum of the breast-height basal areas of trees; TD, tree density; SIN, soil inorganic nitrogen (NH_4_^+^ + NO_3_^–^); POC%, proportion of particulate organic carbon in SOC; LPI%, proportion of labile carbon I in SOC; LPII%, proportion of labile carbon II in SOC; RP% proportion of recalcitrant carbon in SOC; M_*o*_:SOC, mole ratio of poorly crystallized Fe and Al to SOC.

Mantel test showed that soil bacterial β-diversities were significantly associated with MAT, basal area of trees, M_*o*_:SOC, and pH for each soil layer (Table 3). Surface soil β-diversities were also significantly correlated with LPI% and RP%, while deep soil bacterial β-diversities were significantly correlated with tree diversity, soil C content, and POC%. Partial Mantel test further verified the significant associations of MAT and pH with soil bacterial β-diversities in the surface and subsurface layers. In the deep layer, soil bacterial β-diversities had significant partial correlations with MAT, tree diversity and density, SIN, LPI%, and M_*o*_:SOC, but not with soil pH.

### Soil Bacterial Composition in Relation to Environmental Variables

**FIGURE 4:**
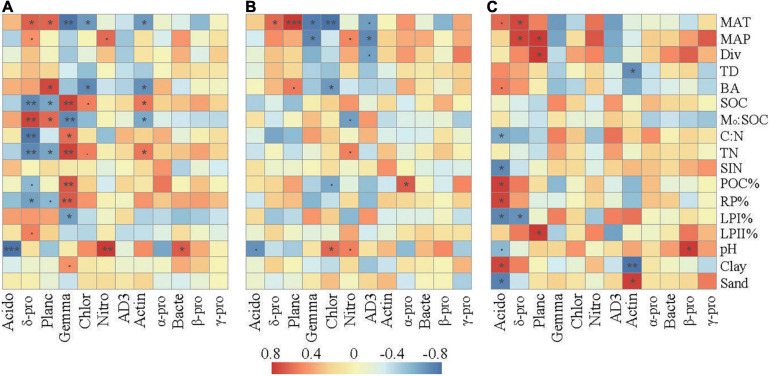
Spearman’s rank correlations between the relative abundances of the dominant bacterial taxa and the selected soil properties in the surface soil layer **(A)**, subsurface soil layer **(B)**, and deep soil layer **(C)**. The correlation coefficients ranging from negative to positive are indicated by color intensity changing from dark blue to red, as illustrated by the figure legend. MAT, mean annual temperature; MAP, mean annual precipitation; Div, Shannon’s index of trees; BA, the sum of breast-height basal areas of trees; TD, tree density; SIN, soil inorganic nitrogen (NH_4_^+^ + NO_3_^–^); POC%, proportion of particulate organic carbon in SOC; LPI%, proportion of labile carbon I in SOC; LPII%, proportion of labile carbon II in SOC; RP% proportion of recalcitrant carbon in SOC; M_*o*_:SOC, mole ratio of poorly crystallized Fe and Al to SOC. Acido, acidobacteria; δ-pro, delta-proteobacteria; Planc, planctomycetes; Gemma, gemmatimonadetes; Chlor, chloroflexi; Nitro, nitrospirae; Actin, actinobacteria; α-pro, alpha-proteobacteria; Bacte, bacteroidetes; β-pro, beta-proteobacteria; γ-pro, gamma-proteobacteria. represents a significant relationship at *p* < 0.1, *p < 0.05; **p < 0.01; ***p < 0.001. *p*-Values were corrected for multiple testing using the Benjamini and Hochberg method.

Correlation analysis revealed that the relative abundances of the dominant taxa were associated with specific climate conditions, vegetation, and soil properties (Figure 4). In the surface layer, the relative abundances of Delta-proteobacteria and Planctomycetes were positively associated with MAT and M_*o*_:SOC and negatively associated with SOC, TN, and RP%. The relative abundances of Gemmatimonadetes, Chloroflexi, and Actinobacteria were negatively associated with MAT and M_*o*_:SOC and positively associated with SOC and TN. Gemmatimonadetes were further positively associated with C:N, POC%, and RP% and negatively associated with LPI%. Soil pH was negatively correlated with Acidobacteria and positively correlated with Nitrospirae and Bacteroidetes.

Compared with those in the surface layer, the relative abundances of these main bacterial taxa in the subsurface and deep layers showed much weaker correlations with the environmental parameters. SOC, TN, C:N, and M_*o*_:SOC showed little relationships with the relative abundances of the dominant bacterial taxa. In the subsurface layer, pH was slightly correlated with Acidobacteria, Chloroflexi, and Nitrospirae. In the deep layer, soil pH was only significantly correlated with the relative abundance of Acidobacteria and Beta-proteobacteria. In addition, Delta-proteobacteria and Planctomycetes were significantly correlated with parameters associated with soil C fractions (POC%, LPI%, LPII%, or RP%) in the deep layer.

At the class level, soil pH was significantly correlated with multiple taxa (belonging to Acidobacteria, Actinobacteria, Bacteroidetes, and Nitrospirae) in the surface and subsurface layers, but these relationships became much weaker or even disappeared in the deep layer (Supplementary Figure 4). SOC properties were significantly correlated with Acidimicrobiia (belonging to Actinobacteria), TK10 (belonging to Chloroflexi), and Gemm-1 (belonging to Gemmatimonadetes).

**FIGURE 5:**
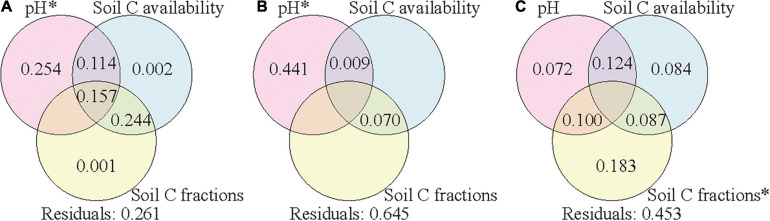
Variation partition analysis of the effects of soil pH, soil C availability, and soil C fractions on soil bacterial community composition in the surface soil layer **(A)**, subsurface soil layer **(B)**, and deep soil layer **(C)**. Values < 0 are not shown. Significant values (*p* < 0.05) after 999 permutations are indicated with an asterisk. Residuals mean the unexplained variation. Soil C availability includes variables of SOC content and the mole ratio of poorly crystallized Fe and Al to SOC (M_*o*_:SOC); soil C factions include variables of the labile carbon pool I (LPI%) and recalcitrant carbon pool (RP%). SOC, soil organic carbon.

Variation partitioning was further applied to assess the relative contributions of climate condition, vegetation properties, and soil properties to the variance of soil bacterial community composition in each soil layer. The explained variances were 71, 56, and 61% in the surface, subsurface, and deep layers, respectively (Supplementary Figure 5). The variances of bacterial community compositions were explained by the combined effects of these factors, and soil properties contributed the most to the variances in all the three layers. To assess the effects of soil properties on soil bacterial community composition, we further separated soil properties into three groups (pH, soil C availability, and soil C fractions) to assess their corresponding contributions. Soil pH, soil C availability, and soil C fractions could explain 74, 36, and 55% of the variance of bacterial community composition in the surface, subsurface, and deep layers, respectively (Figure 5). In the surface and subsurface layers, only soil pH showed significant effects and contributed most to the variance of bacterial community composition. In the deep layer, soil C fractions showed significant effects and contributed most to the variance of bacterial community composition, and the explanatory power of soil pH became insignificant. When climate conditions or vegetation properties were co-analyzed with the three group soil properties (Supplementary Figures 6, 7), the explained variances changed little in all the three layers.

## Discussion

### Effects of pH in Shaping Bacterial Diversity and Composition

Soil bacterial diversity and composition were highly associated with soil pH in both surface and deeper layers and were consistent with previous studies that have shown that pH was a predictor of soil bacterial community structure (Lauber et al., 2009; Shen et al., 2013; Cho et al., 2018; Qi et al., 2018). Bacterial α-diversity was higher in soils close to neutral pH in the medium elevation and showed a hump-shaped relationship with elevation, which supported our first hypothesis. Previous studies have shown that most bacteria taxa have intracellular pH levels close to neutral and that acidic pH conditions will impose strong selective pressures to microbial communities, resulting in specialized lineage loss (Tripathi et al., 2012, 2018). In contrast, neutral pH environments will weaken niche-based exclusion, and the adapted lineages will accumulate in neutral pH environments, resulting in greater diversity.

Since low pH conditions impose a significant physiological constraint on soil microbes, some taxa may tolerate it better and dominate in soils if soil pH falls outside a certain range (Lauber et al., 2009). Conversely, some other taxa are unable to survive and have reduced growth, resulting in the transition of bacterial composition. Class-level taxa of Acidobacteria, Actinobacteria, and Bacteroidetes showed stronger correlations with soil pH than other taxa (Figure 4 and Supplementary Figure 4). These results were consistent with previous studies that have shown that Acidobacteria can tolerate an acid environment and that Actinobacteria and Bacteroidetes are more vulnerable to the changes of soil pH (Lauber et al., 2009; Tripathi et al., 2012; Yang et al., 2018).

A higher temperature together with a humid climate could enhance chemical weathering and leaching, which caused the lowest soil pH in the lowest elevation site (1,571–1,721 m a.s.l.). The coniferous forest in the higher elevation also showed a decreased soil pH due to acidic chemical components (Burgess-Conforti et al., 2019). Thus, soil pH in the medium elevation was close to neutral and consequentially led to the highest bacterial diversity. The lowest soil pH in *Q. aliena* forest also made bacterial communities differ significantly from those in other forests.

### Effects of Soil Organic Carbon Properties in Shaping Bacterial Diversity and Composition

M_*o*_:SOC ratio has been proposed as an important indicator of C availability due to the strong chemical association between SOC and poorly crystallized Fe and Al oxides (Gentsch et al., 2018; Chen et al., 2019). LPII% represents the relative abundance of labile C in total SOC, indicating the pool size of the available C sources (Oades et al., 1970). Since most soil microorganisms rely on SOC decomposition to obtain energy, low content and recalcitrant C sources would limit the survival and maintenance of microorganisms and consequently the community diversity (Delgado-Baquerizo et al., 2016; Ren C. et al., 2018b; Tian et al., 2018). It is notable that the significant effect of available C sources on diversity was not observed in the surface layer where SOC was relatively rich. According to the global data from all continents, Delgado-Baquerizo et al. (2016) also found a stronger relationship between C content and bacterial diversity under low C conditions than under rich C conditions. These results suggest that SOC properties may only affect the diversity of bacteria in C-poor situations.

Soil C availability and soil C fractions can also affect bacterial composition due to different life strategies and resource preferences of particular bacterial taxa (Ding et al., 2015; Delgado-Baquerizo et al., 2016; Tian et al., 2018; Shao et al., 2019). The dominant taxa at the genus level belonging to Acidobacteria, Delta-proteobacteria, Planctomycetes, Gemmatimonadetes, Chloroflexi, and Nitrospirae have a lower rRNA operon copy number (Supplementary Table 7), while taxa at the genus level belonging to Actinobacteria, Bacteroidetes, Firmicutes, and Beta- and Gamma-proteobacteria have a higher rRNA operon copy number, suggesting their relatively oligotrophic and copiotrophic life strategies, respectively (Nemergut et al., 2016; Roller et al., 2016). Similarly, Acidobacteria, Delta-proteobacteria, Planctomycetes, Gemmatimonadetes, and Chloroflexi have been generally classified as oligotrophic bacterial taxa and dominate in soils with lower C availability, while Actinobacteria, Bacteroidetes, Beta-proteobacteria, and Gamma-proteobacteria are classified as copiotrophic bacterial taxa and flourish in soils with greater C pools (Fierer et al., 2007; DeBruyn et al., 2011; Yao et al., 2017; Razanamalala et al., 2018; Brewer et al., 2019). The transition from low to high soil C availability could explain the transition from abundant oligotrophic Delta-proteobacteria and Planctomycetes to abundant copiotrophic Actinobacteria along the elevational gradient. In addition, the abundances of oligotrophic Gemmatimonadetes and Chloroflexi were positively correlated with soil C availability for surface soils, which was contrary to what their life strategies would predict. Previous studies have also suggested that Gemmatimonadetes and Chloroflexi can degrade recalcitrant compounds (Wilms et al., 2006; Pascault et al., 2013; Chen et al., 2015; Deng et al., 2019). In this study, Gemmatimonadetes (Gemm-1 at the class level) and Chloroflexi (TK10 at the class level) were positively correlated with RP% (i.e., aromatic C fraction), which suggests that resource preferences can partly explain the variations in the abundances of Gemmatimonadetes and Chloroflexi.

In the deep layer, the relative abundances of the main taxa had little correlations with soil C availability but showed significant correlations with parameters associated with soil C fractions (Figure 4 and Supplementary Figure 4). Previous studies have found that Acidobacteria and Delta-proteobacteria can degrade more recalcitrant substrates (Pascault et al., 2013; Chen et al., 2015), while Planctomycetes can degrade chitin and utilize *N*-acetylglucosamine as a sole C source (Ivanova et al., 2018; Ravin et al., 2018). The significant correlations between LPI% (or LPII%) and the relative abundances of these taxa suggest that resource preferences could explain the transitions of these taxa along the elevational gradient in the deep layer.

In this study, the low decay conditions (lower temperature and litter quality) at high elevation could promote soil C accumulation, especially recalcitrant plant-derived C (i.e., lignin) accumulation (Craig et al., 2018). In contrast, the fast decay conditions (higher temperature and litter quality) at low elevation could reduce soil C accumulation (Prietzel and Christophel, 2014) but promote labile microbial-derived C (i.e., polysaccharides) accumulation (Yang et al., 2020). In this way, soils from medium elevations had higher SOC content and labile C fractions and higher bacterial diversity. The distinct soil C content and fractions among sites also made microbial composition differ significantly.

### Effects of Vegetation Properties and Climate on Bacterial Diversity and Composition

Plant biomass and diversity can regulate bacterial diversity and composition via the supply of C resource and modification of the physical microhabitats and environmental conditions (Mitchell et al., 2010; Prober et al., 2015; Wang et al., 2016; Ren B. et al., 2018a). MAT and MAP can regulate bacterial diversity and composition directly via speciation, competition, and dispersal of microbial communities or indirectly via mediating other environmental factors (Singh et al., 2014; Delgado-Baquerizo et al., 2016; Tian et al., 2018). In this study, the indices of bacterial diversity were significantly correlated with MAP and tree diversity in the deep soil layer (Table 4). However, the significant correlations disappeared when variations of M_*o*_:SOC, LPII%, and pH were controlled (Supplementary Table 6). These results suggest that MAP and tree diversity might indirectly affect bacterial diversity in the deep layer through their effect on soil properties, especially SOC factions (Supplementary Table 4). However, these effects were not observed in the surface layer. This might be because surface soil can be strongly influenced by some additional biogeochemical processes such as bioturbation (Wilkinson et al., 2009), and the effects of vegetation properties and climate on bacterial diversity might be masked by some other biotic and abiotic factors.

Variation in soil bacterial composition reflected changes in vegetation biomass and MAT in all the three soil layers (Table 4), suggesting that quantity of C source and ecological processes related to temperature may play an important role in structuring bacterial biodiversity. In the deep layer, tree diversity also contributed to the variation of bacterial composition. These results indicate that climate and vegetation can affect bacterial communities in the deep soil layer and can potentially affect soil biogeochemical processes.

### Determining Factors of Soil Bacterial Community Composition

The variances of bacterial community composition were highly explained by the combined effects of climate conditions, vegetation properties, and soil properties. Among them, soil properties contributed the most in all the three soil layers (Supplementary Figure 5). Inclusion of climate conditions or vegetation properties in the models with the three group of soil properties had little effect on the explanatory power (Supplementary Figure 6, 7). These results suggest that bacterial community composition was directly regulated by soil properties, while climate and vegetation probably regulated bacterial community composition through their associations with soil pH, soil C availability, and soil C fractions.

Among the soil properties, pH contributed the most to the variance of bacterial community composition in the surface and subsurface soil layers, while soil C fractions contributed the most to the variance in the deep layer (Figure 5). This result supports our second hypothesis that driving factors of the elevational patterns of soil bacterial communities differed across soil layers. Soil pH has been widely reported as the driving factor of soil bacterial communities for surface soils (Lauber et al., 2009; Siles and Margesin, 2016; Xia et al., 2016). Soil pH in the deep layer was closer to neutral than that in the surface layer (Table 2), which might decrease the selective pressures and consequently reduce the effect of pH. The weaker effect of pH was also validated by the results of the partial Mantel test in the deep layer (Table 3). In addition, low C availability in the deep layer would limit the substrate quantity and consequently the substrate diversity in chemical structures. In this case, the bacterial taxa could be filtered by the specific types of substrates according to their resource preferences (Paterson et al., 2008; Di Lonardo et al., 2017), and the composition of bacterial community was more driven by soil C fractions. In contrast, when C availability was high with great substrate diversity in chemical structures (i.e., surface layer soils), soil bacterial taxa could utilize various types of substrates due to their plasticity in C use (Morrissey et al., 2017); thus, soil C fractions might have a small effect on the composition of bacterial communities.

It was notable that the sampling sizes (*n* = 15 for each soil layer) were relatively small, which might limit the reliability of our results. A larger number of samples would provide more concrete results to better understand the elevational patterns of soil microbial community and the driving factors in different soil layers.

## Conclusion

This study demonstrated that soil bacterial diversity and composition shifted along the elevational gradient in both surface and deep layers, but the driving factors differed across soil layers. Soil pH was the main factor determining the elevational patterns of surface soil bacterial diversity and community composition, while deep soil bacterial diversity and composition were more explained by soil C availability and C fractions. Climate and forest transitions along the elevational gradient could modify soil pH, C availability, and C fractions, which then drove the variations in soil bacterial diversity and community composition. These findings will improve our understanding of the relationships between soil C cycling and bacterial communities and will potentially help us better predict soil responses to future environmental changes.

## Data Availability Statement

The datasets presented in this study can be found in online repositories. The names of the repository/repositories and accession number(s) can be found in the article/Supplementary Material.

## Author Contributions

QT, YW, and FL designed the study. YT and YW conducted sample collection. QT and YJ performed the experiments for environmental parameters and data analyses, and drafted the manuscript. All authors contributed to the final version of the manuscript.

## Conflict of Interest

The authors declare that the research was conducted in the absence of any commercial or financial relationships that could be construed as a potential conflict of interest.

## Publisher’s Note

All claims expressed in this article are solely those of the authors and do not necessarily represent those of their affiliated organizations, or those of the publisher, the editors and the reviewers. Any product that may be evaluated in this article, or claim that may be made by its manufacturer, is not guaranteed or endorsed by the publisher.

## Footnotes

1
https://www.arb-silva.de/

2
http://qiime.org/tutorials/tutorial.html

3
http://sourceforge.net/projects/rdp-classifier/

4
https://www.ncbi.nlm.nih.gov/

5
http://www.rproject.org/
