# Supplementary material for: Soil pH and Organic Carbon Properties Drive Soil Bacterial Communities in Surface and Deep Layers Along an Elevational Gradient
Source: Front Microbiol. 2021 Jul 30;12:646124. doi: 10.3389/fmicb.2021.646124 (PMC8363232; doi:10.3389/fmicb.2021.646124)
Supplement: Supplementary file 2 [file Data_Sheet_2.docx]

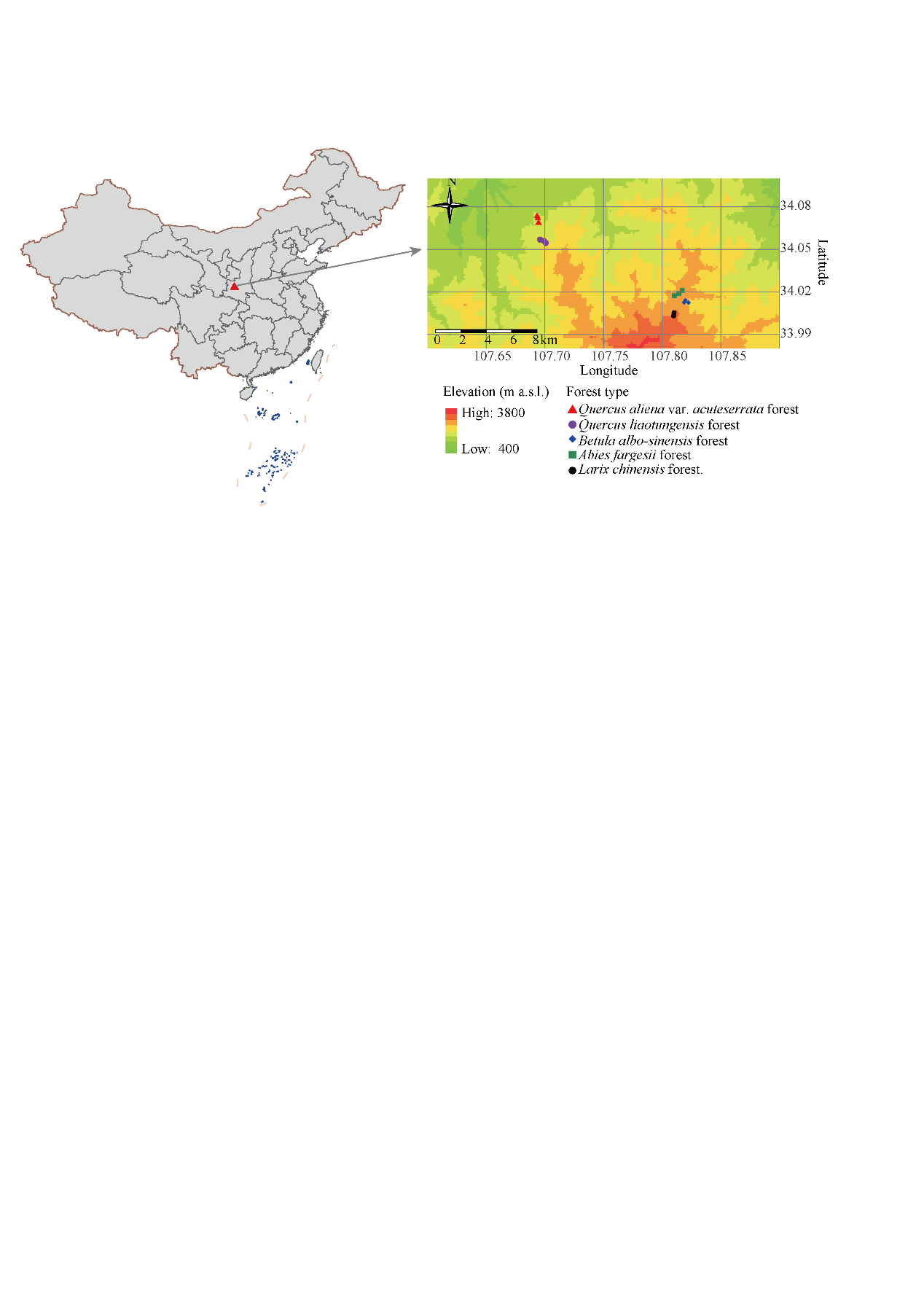


**Figure S1** Location and the topographic image of the studying sites on the northern slope in Taibai Mountain.


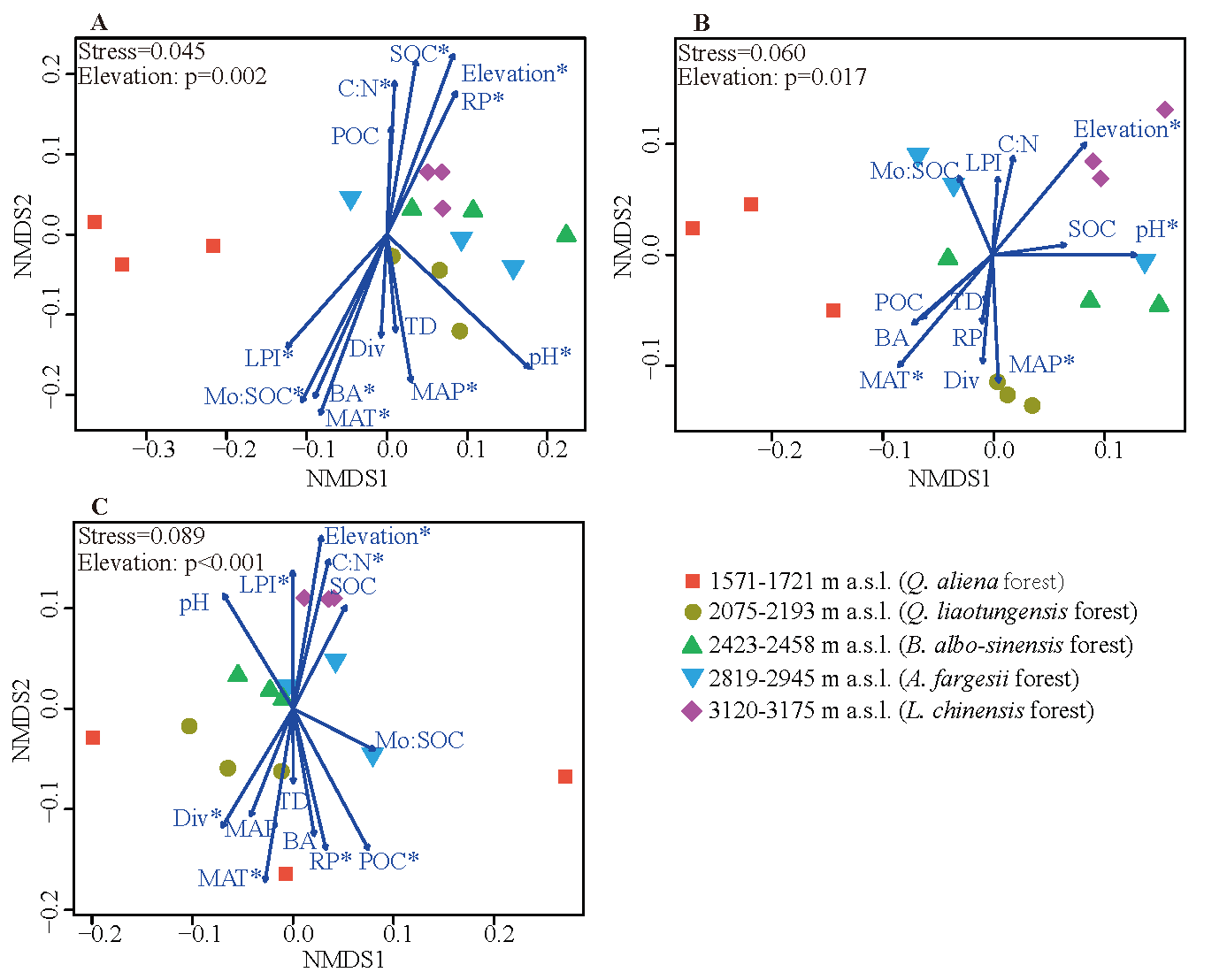


**Figure S2** Non-metric multidimensional scaling (NMDS) ordinations of soil bacterial community composition based on weighted UniFrac distances derived from OTU composition in surface soil layer (A), subsurface soil layer (B) and deep soil layer (C). Vectors represent the strength/direction of the weight of environmental variables on bacterial communities. Significant correlations between ordinations and environmental factors are indicated with asterisks (adjusted p < 0.05). P-values were adjusted according to the false discovery rate procedure of Benjamini and Hochberg. MAT, mean annual temperature; MAP, mean annual precipitation; Div, Shannon index of trees; BA, the sum of breast-height basal areas of trees; TD, tree density; POC%, proportion of particulate organic carbon in SOC; LPI%, proportion of labile carbon I in SOC; LPII %, proportion of labile carbon II in SOC; RP% proportion of recalcitrant carbon in SOC; M_o_:SOC, mole ratio of poorly crystallized Fe and Al to SOC.


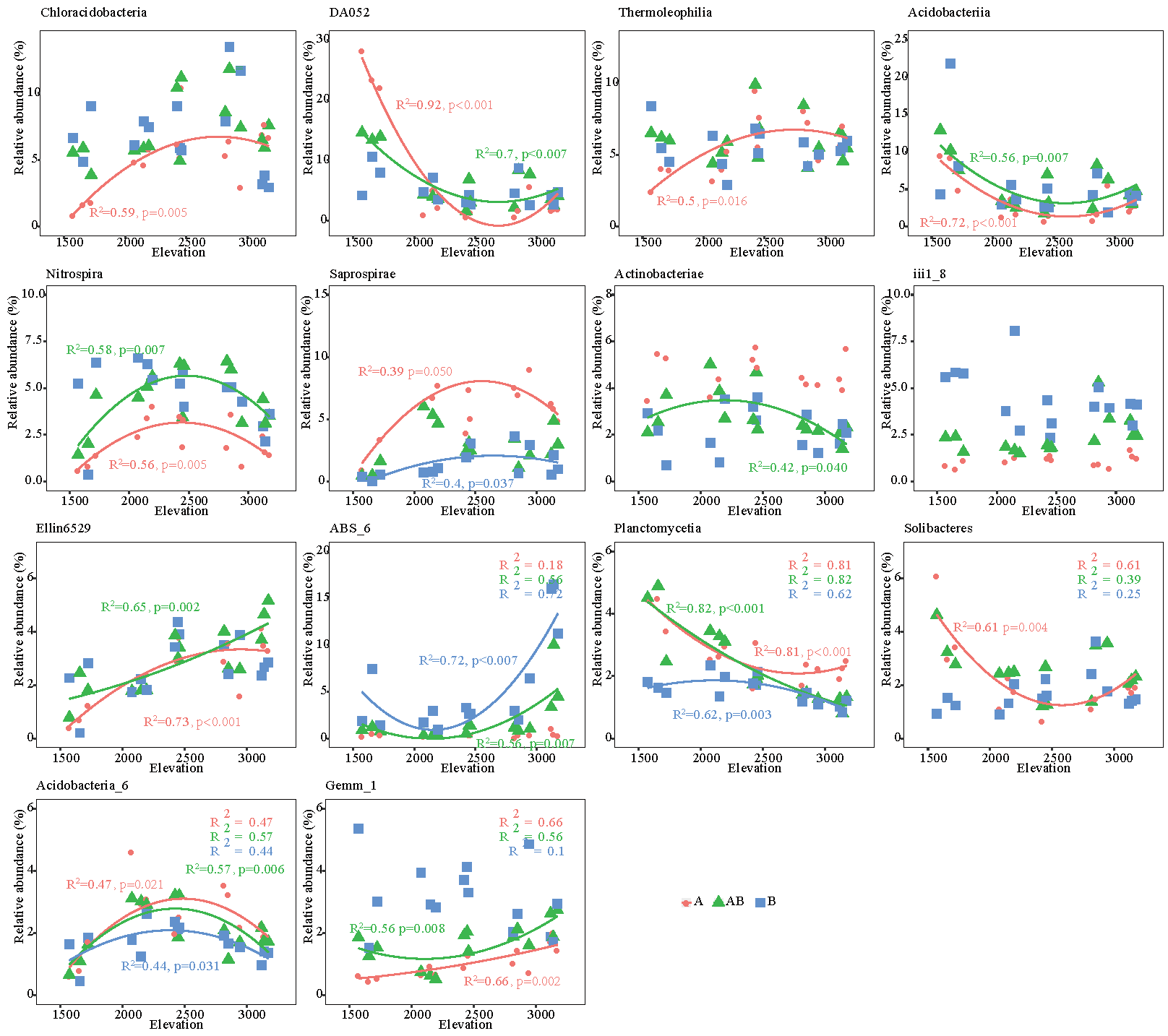


**Figure S3** Relative abundances of the dominant bacterial taxa (at class level) along the elevational gradient. Lines in each plot represent the least squares regression fits.


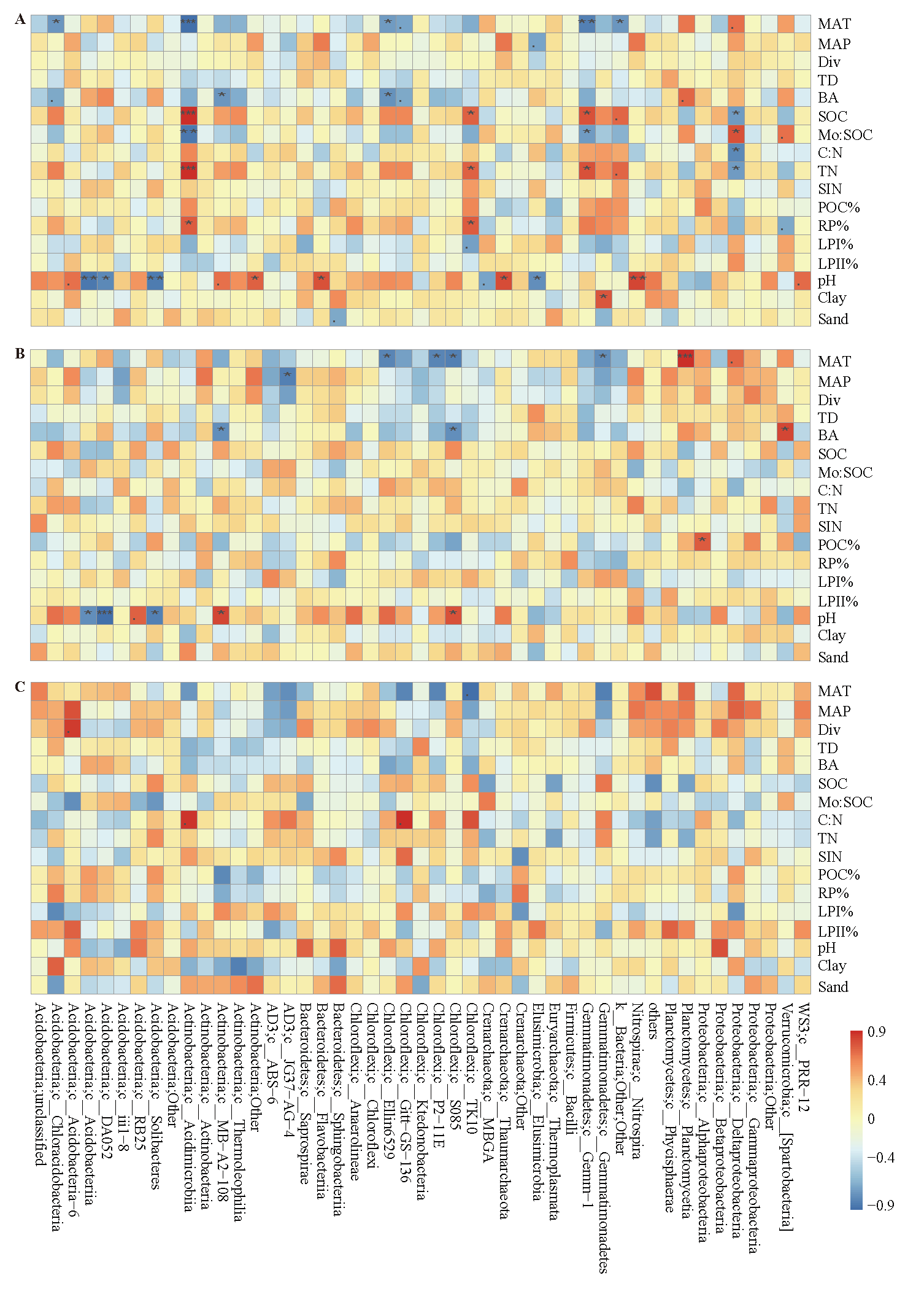
**Figure S4** Spearman’s rank correlations between the relative abundances of the dominant bacterial taxa and the selected soil properties in surface soil layer (A), subsurface soil layer (B) and deep soil layer (C). The correlation coefficients ranging from negative to positive are indicated by color intensity changing from dark blue to red, as illustrated by the figure legend. • represents a significant relationship at p<0.1, * represents p<0.05, ** represents p<0.01, and *** represents p<0.001. P-values were corrected for multiple testing using the Benjamini and Hochberg method.


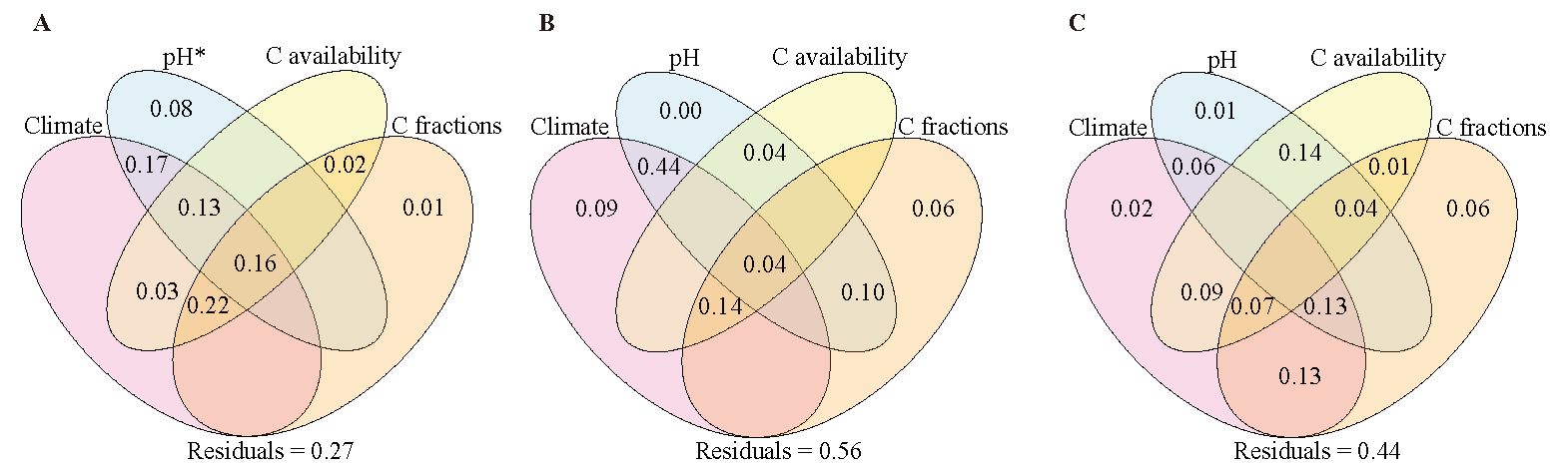
**
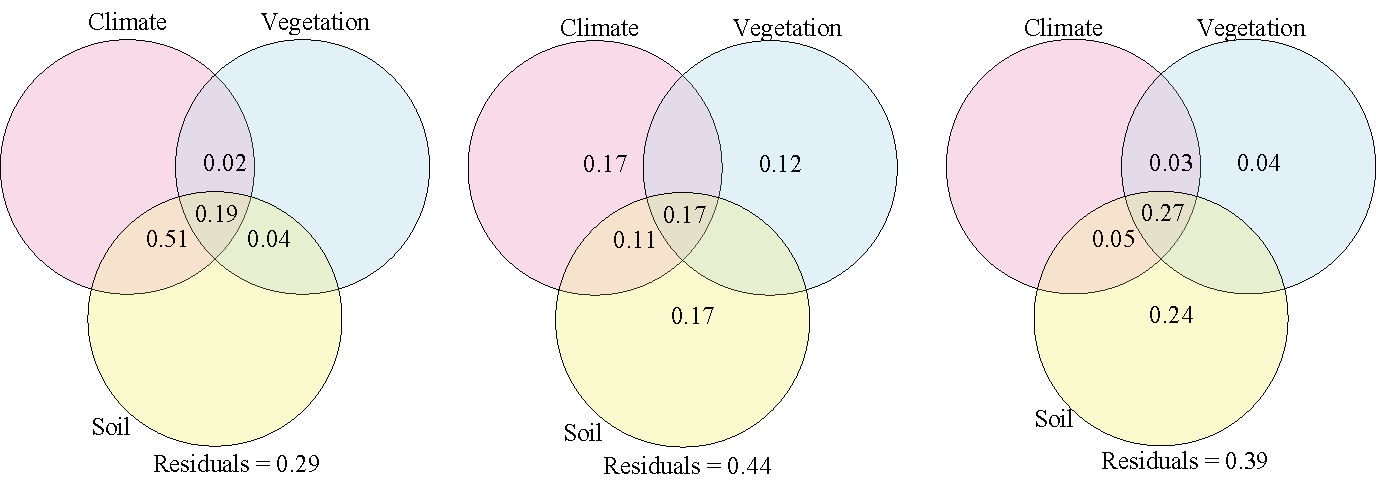
**

**Figure S5** Variation partition analysis of the effects of climate conditions, vegetation properties and soil properties on soil bacterial community composition in surface soil layer (A), subsurface soil layer (B) and deep soil layer (C). Values <0 are not shown. Significant values (p < 0.05) after 999 permutations are indicated with asterisk. Residuals mean the unexplained variation. Climate conditions include variables of mean annual temperature (MAT) and mean annual precipitation (MAP); Vegetation properties include variables of Shannon index and basal area of the trees; soil properties include variables of pH, soil organic carbon (SOC) content and the mole ratio of poorly crystallized Fe and Al to SOC (M_o_:SOC); labile carbon pool I (LPI%) and recalcitrant carbon pool (RP%).


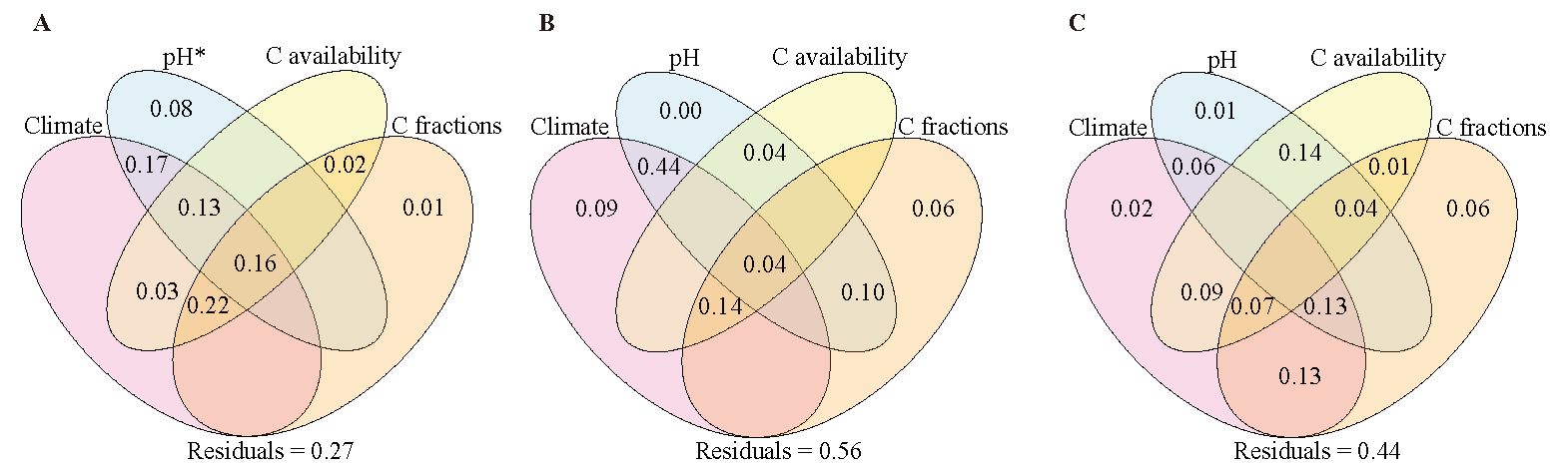


**Figure S6** Variation partition analysis of the effects of climate conditions, soil pH, soil C availability and soil C fractions on soil bacterial community composition in surface soil layer (A), subsurface soil layer (B) and deep soil layer (C). Values <0 are not shown. Significant values (p < 0.05) after 999 permutations are indicated with asterisk. Residuals mean the unexplained variation. Climate conditions include variables of mean annual temperature (MAT) and mean annual precipitation (MAP); soil C availability include variables of soil organic carbon (SOC) content and the mole ratio of poorly crystallized Fe and Al to SOC (M_o_:SOC); soil C factions include variables of labile carbon pool I (LPI%) and recalcitrant carbon pool (RP%).


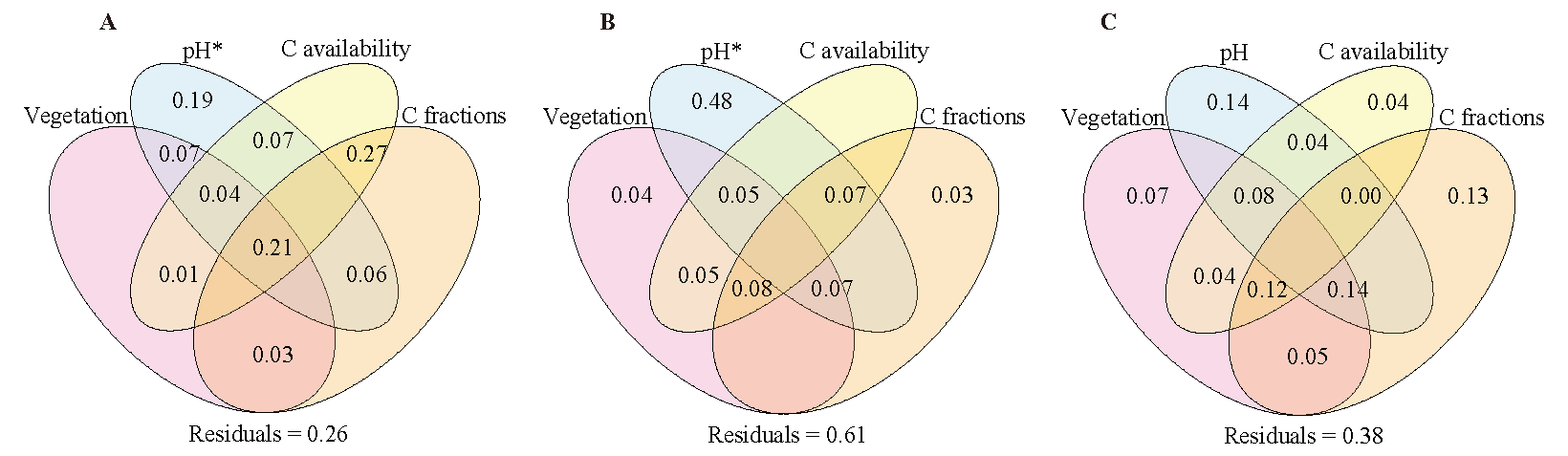


**Figure S7** Variation partition analysis of the effects of vegetation properties, soil pH, soil C availability and soil C fractions on soil bacterial community composition in surface soil layer (A), subsurface soil layer (B) and deep soil layer (C). Values <0 are not shown. Significant values (p < 0.05) after 999 permutations are indicated with asterisk. Residuals mean the unexplained variation. Vegetation properties include variables of Shannon index and basal area of the trees; soil C availability include variables of soil organic carbon (SOC) content and the mole ratio of poorly crystallized Fe and Al to SOC (M_o_:SOC); soil C factions include variables of labile carbon pool I (LPI%) and recalcitrant carbon pool (RP%).

**Table S1** The geographic distances (km) between the sampling plots.

|  | Qa-plot1 | Qa-plot2 | Qa-plot3 | Ql-plot1 | Ql-plot2 | Ql-plot3 | Ba-plot1 | Ba-plot2 | Ba-plot3 | Af-plot1 | Af-plot2 | Af-plot3 | Lc-plot1 | Lc-plot2 |  |
| --- | --- | --- | --- | --- | --- | --- | --- | --- | --- | --- | --- | --- | --- | --- | --- |
| Qa-plot1 |  |  |  |  |  |  |  |  |  |  |  |  |  |  |  |
| Qa-plot2 | 0.08 |  |  |  |  |  |  |  |  |  |  |  |  |  |  |
| Qa-plot3 | 0.20 | 0.12 |  |  |  |  |  |  |  |  |  |  |  |  |  |
| Ql-plot1 | 0.62 | 0.54 | 0.42 |  |  |  |  |  |  |  |  |  |  |  |  |
| Ql-plot2 | 0.88 | 0.80 | 0.68 | 0.39 |  |  |  |  |  |  |  |  |  |  |  |
| Ql-plot3 | 1.04 | 0.96 | 0.84 | 0.56 | 0.17 |  |  |  |  |  |  |  |  |  |  |
| Ba-plot1 | 14.18 | 14.11 | 14.03 | 13.84 | 13.46 | 13.29 |  |  |  |  |  |  |  |  |  |
| Ba-plot2 | 14.47 | 14.40 | 14.32 | 14.14 | 13.75 | 13.58 | 0.30 |  |  |  |  |  |  |  |  |
| Ba-plot3 | 14.09 | 14.02 | 13.94 | 13.75 | 13.36 | 13.19 | 0.11 | 0.39 |  |  |  |  |  |  |  |
| Af-plot1 | 13.13 | 13.06 | 12.98 | 12.79 | 12.41 | 12.24 | 1.05 | 1.34 | 0.96 |  |  |  |  |  |  |
| Af-plot2 | 13.55 | 13.48 | 13.40 | 13.21 | 12.83 | 12.66 | 0.63 | 0.93 | 0.55 | 0.43 |  |  |  |  |  |
| Af-plot3 | 13.86 | 13.79 | 13.71 | 13.53 | 13.15 | 12.98 | 0.37 | 0.65 | 0.34 | 0.76 | 0.33 |  |  |  |  |
| Lc-plot1 | 13.18 | 13.11 | 13.03 | 12.83 | 12.44 | 12.27 | 1.10 | 1.37 | 0.99 | 0.41 | 0.64 | 0.93 |  |  |  |
| Lc-plot2 | 13.17 | 13.10 | 13.02 | 12.82 | 12.43 | 12.26 | 1.11 | 1.38 | 1.00 | 0.41 | 0.65 | 0.94 | 0.01 |  |  |
| Lc-plot3 | 13.14 | 13.06 | 12.98 | 12.78 | 12.40 | 12.22 | 1.17 | 1.44 | 1.07 | 0.49 | 0.73 | 1.02 | 0.10 | 0.09 |  |

Qa represents *Quercus aliena* var. *acuteserrata* forest; Ql represents *Quercus liaotungensis* forest; Ba represents *Betula albo-sinensis* forest; Af represents *Abies fargesii* forest; Lc represents *Larix chinensis* forest.

**Table S2** The correlation matrix (Spearman’s rank correlations) between environmental characteristics for mineral A layer.

|  | Ele | MAT | MAP | Div | TD | BA | SOC | M_o_:SOC | C:N | TN | SIN | POC% | RP% | LPI% | LPII% | pH | Clay | Sand |
| --- | --- | --- | --- | --- | --- | --- | --- | --- | --- | --- | --- | --- | --- | --- | --- | --- | --- | --- |
| Ele |  |  |  |  |  |  |  |  |  |  |  |  |  |  |  |  |  |  |
| MAT | **-1** |  |  |  |  |  |  |  |  |  |  |  |  |  |  |  |  |  |
| MAP | -0.382 | 0.382 |  |  |  |  |  |  |  |  |  |  |  |  |  |  |  |  |
| Div | -0.254 | 0.254 | **0.807** |  |  |  |  |  |  |  |  |  |  |  |  |  |  |  |
| TD | -0.239 | 0.239 | 0.129 | 0.221 |  |  |  |  |  |  |  |  |  |  |  |  |  |  |
| BA | -0.579 | 0.579 | -0.011 | -0.025 | **0.642** |  |  |  |  |  |  |  |  |  |  |  |  |  |
| SOC | **0.939** | **-0.939** | -0.288 | -0.14 | -0.322 | -0.564 |  |  |  |  |  |  |  |  |  |  |  |  |
| M_o_:SOC | **-0.875** | **0.875** | 0.282 | 0.133 | 0.390 | 0.586 | **-0.971** |  |  |  |  |  |  |  |  |  |  |  |
| C:N | **0.686** | **-0.686** | **-0.715** | -0.466 | -0.198 | -0.211 | **0.725** | **-0.729** |  |  |  |  |  |  |  |  |  |  |
| TN | **0.939** | **-0.939** | -0.288 | -0.140 | -0.322 | -0.564 | **1** | **-0.971** | **0.725** |  |  |  |  |  |  |  |  |  |
| SIN | 0.211 | -0.211 | -0.098 | 0.269 | -0.218 | -0.029 | 0.207 | -0.154 | 0.204 | 0.207 |  |  |  |  |  |  |  |  |
| POC% | 0.604 | -0.604 | -0.466 | -0.405 | -0.206 | -0.089 | **0.711** | **-0.657** | **0.750** | **0.711** | 0.175 |  |  |  |  |  |  |  |
| RP% | **0.754** | **-0.754** | -0.164 | -0.100 | -0.363 | -0.486 | **0.879** | **-0.907** | **0.536** | **0.879** | 0.132 | **0.664** |  |  |  |  |  |  |
| LPI% | **-0.704** | **0.704** | 0.043 | -0.104 | 0.014 | 0.268 | **-0.818** | **0.800** | -0.500 | **-0.818** | -0.204 | **-0.639** | **-0.896** |  |  |  |  |  |
| LPII% | -0.371 | 0.371 | 0.245 | 0.294 | **0.704** | 0.436 | -0.464 | **0.557** | -0.293 | -0.464 | -0.014 | -0.289 | -0.496 | 0.146 |  |  |  |  |
| pH | 0.191 | -0.191 | 0.580 | 0.287 | -0.115 | -0.465 | 0.166 | -0.129 | -0.368 | 0.166 | -0.32 | -0.173 | 0.206 | -0.214 | 0.113 |  |  |  |
| Clay | 0.200 | -0.200 | -0.113 | -0.341 | 0.354 | 0.064 | 0.182 | -0.161 | 0.225 | 0.182 | -0.425 | 0.375 | 0.296 | -0.361 | 0.257 | 0.080 |  |  |
| Sand | -0.122 | 0.122 | 0.244 | 0.276 | -0.47 | -0.324 | -0.127 | 0.063 | -0.322 | -0.127 | 0.166 | -0.461 | -0.223 | 0.449 | -0.492 | 0.041 | -0.772 |  |

Ele, elevation; MAT, mean annual temperature; MAP, mean annual precipitation; Div, Shannon index of trees; BA, the sum of breast-height basal areas of trees; TD, tree density; SIN, soil inorganic nitrogen (NH_4_^+^+NO_3_^-^); POC%, proportion of particulate organic carbon in SOC; LPI%, proportion of labile carbon I in SOC; LPII %, proportion of labile carbon II in SOC; RP% proportion of recalcitrant carbon in SOC; M_o_:SOC, mole ratio of poorly crystallized Fe and Al to SOC. Numbers in bold indicate significant correlations with *p* < 0.05. P-values were corrected for multiple testing using the Benjamini and Hochberg method.

**Table S3** The correlation matrix (Spearman’s rank correlations) between environmental characteristics for mineral AB layer.

|  | Ele | MAT | MAP | Div | TD | BA | SOC | M_o_:SOC | C:N | TN | SIN | POC% | RP% | LPI% | LPII% | pH | Clay | Sand |
| --- | --- | --- | --- | --- | --- | --- | --- | --- | --- | --- | --- | --- | --- | --- | --- | --- | --- | --- |
| Ele |  |  |  |  |  |  |  |  |  |  |  |  |  |  |  |  |  |  |
| MAT | **-1** |  |  |  |  |  |  |  |  |  |  |  |  |  |  |  |  |  |
| MAP | -0.382 | 0.382 |  |  |  |  |  |  |  |  |  |  |  |  |  |  |  |  |
| Div | -0.254 | 0.254 | **0.807** |  |  |  |  |  |  |  |  |  |  |  |  |  |  |  |
| TD | -0.239 | 0.239 | 0.129 | 0.221 |  |  |  |  |  |  |  |  |  |  |  |  |  |  |
| BA | -0.579 | 0.579 | -0.011 | -0.025 | 0.642 |  |  |  |  |  |  |  |  |  |  |  |  |  |
| SOC | 0.461 | -0.461 | 0.266 | 0.376 | 0.254 | -0.493 |  |  |  |  |  |  |  |  |  |  |  |  |
| M_o_:SOC | 0.032 | -0.032 | -0.615 | **-0.731** | -0.35 | 0.196 | **-0.775** |  |  |  |  |  |  |  |  |  |  |  |
| C:N | 0.450 | -0.450 | -0.168 | -0.022 | 0.102 | -0.232 | 0.579 | -0.264 |  |  |  |  |  |  |  |  |  |  |
| TN | 0.300 | -0.300 | 0.386 | 0.405 | 0.298 | -0.425 | **0.929** | **-0.821** | 0.318 |  |  |  |  |  |  |  |  |  |
| SIN | 0.025 | -0.025 | -0.166 | -0.014 | -0.477 | -0.364 | -0.157 | 0.007 | -0.421 | -0.032 |  |  |  |  |  |  |  |  |
| POC% | -0.468 | 0.468 | 0.411 | 0.425 | 0.499 | 0.574 | -0.071 | -0.218 | 0.039 | 0.023 | -0.459 |  |  |  |  |  |  |  |
| RP% | -0.507 | 0.507 | 0.332 | 0.176 | 0.458 | 0.386 | 0.175 | -0.454 | 0.121 | 0.275 | -0.061 | 0.286 |  |  |  |  |  |  |
| LPI% | 0.407 | -0.407 | -0.497 | -0.323 | -0.597 | -0.361 | -0.346 | 0.639 | -0.186 | -0.450 | 0.304 | -0.363 | **-0.879** |  |  |  |  |  |
| LPII% | 0.143 | -0.143 | 0.447 | 0.452 | 0.495 | 0.100 | 0.296 | -0.343 | 0.011 | 0.329 | -0.475 | 0.382 | -0.132 | -0.25 |  |  |  |  |
| pH | 0.575 | -0.575 | 0.229 | 0.172 | -0.136 | -0.536 | 0.646 | -0.350 | 0.418 | 0.518 | -0.039 | -0.400 | -0.061 | -0.061 | 0.125 |  |  |  |
| Clay | -0.154 | 0.154 | 0.300 | 0.244 | **0.708** | 0.346 | 0.371 | -0.468 | 0.254 | 0.411 | -0.661 | 0.651 | 0.393 | -0.621 | 0.568 | -0.111 |  |  |
| Sand | 0.211 | -0.211 | -0.136 | -0.133 | **-0.717** | -0.486 | -0.186 | 0.279 | -0.321 | -0.182 | **0.682** | **-0.731** | -0.368 | 0.539 | -0.507 | 0.257 | **-0.943** |  |

Ele, elevation; MAT, mean annual temperature; MAP, mean annual precipitation; Div, Shannon index of trees; BA, the sum of breast-height basal areas of trees; TD, tree density; SIN, soil inorganic nitrogen (NH_4_^+^+NO_3_^-^); POC%, proportion of particulate organic carbon in SOC; LPI%, proportion of labile carbon I in SOC; LPII %, proportion of labile carbon II in SOC; RP% proportion of recalcitrant carbon in SOC; M_o_:SOC, mole ratio of poorly crystallized Fe and Al to SOC. Numbers in bold indicate significant correlations with *p* < 0.05. P-values were corrected for multiple testing using the Benjamini and Hochberg method.

**Table S4** The correlation matrix (Spearman’s rank correlations) between environmental characteristics for mineral B layer.

|  | Ele | MAT | MAP | Div | TD | BA | SOC | M_o_:SOC | C:N | TN | SIN | POC% | RP% | LPI% | LPII% | pH | Clay | Sand |
| --- | --- | --- | --- | --- | --- | --- | --- | --- | --- | --- | --- | --- | --- | --- | --- | --- | --- | --- |
| Ele |  |  |  |  |  |  |  |  |  |  |  |  |  |  |  |  |  |  |
| MAT | **-1** |  |  |  |  |  |  |  |  |  |  |  |  |  |  |  |  |  |
| MAP | -0.382 | 0.382 |  |  |  |  |  |  |  |  |  |  |  |  |  |  |  |  |
| Div | -0.254 | 0.254 | **0.807** |  |  |  |  |  |  |  |  |  |  |  |  |  |  |  |
| TD | -0.239 | 0.239 | 0.129 | 0.221 |  |  |  |  |  |  |  |  |  |  |  |  |  |  |
| BA | -0.579 | 0.579 | -0.011 | -0.025 | **0.642** |  |  |  |  |  |  |  |  |  |  |  |  |  |
| SOC | **0.725** | **-0.725** | -0.038 | -0.018 | -0.05 | -0.521 |  |  |  |  |  |  |  |  |  |  |  |  |
| M_o_:SOC | -0.254 | 0.254 | **-0.665** | -0.613 | 0.082 | 0.518 | **-0.632** |  |  |  |  |  |  |  |  |  |  |  |
| C:N | **0.770** | **-0.770** | -0.161 | -0.095 | -0.449 | -0.599 | 0.554 | -0.336 |  |  |  |  |  |  |  |  |  |  |
| TN | **0.664** | **-0.664** | 0.002 | -0.029 | 0.002 | -0.446 | **0.971** | -0.625 | 0.42 |  |  |  |  |  |  |  |  |  |
| SIN | **0.654** | **-0.654** | -0.059 | -0.168 | -0.433 | -0.579 | 0.232 | -0.225 | **0.652** | 0.179 |  |  |  |  |  |  |  |  |
| POC% | -0.482 | 0.482 | 0.552 | 0.398 | 0.273 | 0.436 | -0.039 | -0.271 | -0.239 | 0.011 | -0.561 |  |  |  |  |  |  |  |
| RP% | -0.243 | 0.243 | 0.214 | 0.168 | 0.252 | 0.189 | 0.279 | -0.286 | -0.306 | 0.311 | -0.525 | **0.675** |  |  |  |  |  |  |
| LPI% | 0.411 | -0.411 | -0.520 | -0.419 | -0.416 | -0.307 | -0.150 | 0.436 | 0.450 | -0.236 | 0.525 | **-0.739** | **-0.850** |  |  |  |  |  |
| LPII% | -0.207 | 0.207 | **0.667** | 0.576 | 0.301 | 0.018 | -0.046 | -0.531 | -0.314 | 0.036 | 0.007 | 0.281 | 0.071 | -0.474 |  |  |  |  |
| pH | 0.475 | -0.475 | 0.368 | 0.362 | -0.161 | -0.568 | 0.307 | -0.568 | 0.402 | 0.321 | 0.579 | -0.196 | -0.218 | 0.043 | 0.415 |  |  |  |
| Clay | -0.246 | 0.246 | 0.143 | 0.219 | **0.702** | 0.486 | 0.218 | -0.107 | -0.327 | 0.218 | -0.564 | 0.500 | 0.611 | **-0.643** | 0.168 | -0.45 |  |  |
| Sand | 0.236 | -0.236 | -0.038 | -0.133 | **-0.735** | -0.518 | -0.193 | 0.025 | 0.384 | -0.207 | **0.689** | -0.561 | **-0.668** | **0.646** | -0.177 | 0.446 | **-0.929** |  |

Ele, elevation; MAT, mean annual temperature; MAP, mean annual precipitation; Div, Shannon index of trees; BA, the sum of breast-height basal areas of trees; TD, tree density; SIN, soil inorganic nitrogen (NH_4_^+^+NO_3_^-^); POC%, proportion of particulate organic carbon in SOC; LPI%, proportion of labile carbon I in SOC; LPII %, proportion of labile carbon II in SOC; RP% proportion of recalcitrant carbon in SOC; M_o_:SOC, mole ratio of poorly crystallized Fe and Al to SOC. Numbers in bold indicate significant correlations with *p* < 0.05. P-values were corrected for multiple testing using the Benjamini and Hochberg method.

**Table S5** Effects of elevation and soil layer on soil bacterial community diversity and composition by using linear mixed models. P value of the linear mixed models show in the table.

|  | Elevation | Layer | Elevation*layer |
| --- | --- | --- | --- |
| Observed OTUs | 0.015 | 0 | 0.023 |
| Shannon’s diversity | 0.008 | 0 | 0.012 |
| Faith's phylogenetic diversity | 0.007 | 0 | 0.012 |
| Weighted mean operon copy number | 0.439 | 0.149 | 0.007 |
| *Acidobacteria* | 0 | 0.015 | 0.016 |
| *Delta-proteobacteria* | 0 | 0 | 0.006 |
| *Planctomycetes* | 0 | 0 | 0 |
| *Gemmatimonadetes* | 0 | 0 | 0.016 |
| *Chloroflexi* | 0 | 0 | 0 |
| *Nitrospirae* | 0.009 | 0 | 0.259 |
| *AD3* | 0 | 0 | 0 |
| *Actinobacteria* | 0.004 | 0.006 | 0.015 |
| *Alpha-proteobacteria* | 0.536 | 0 | 0 |
| *Bacteroidetes* | 0.019 | 0 | 0 |
| *Beta-proteobacteria* | 0 | 0.195 | 0.27 |
| *Gamma-proteobacteria* | 0.156 | 0 | 0.147 |
|  |  |  |  |
| *Chloracidobacteria* (*Acidobacteria*) | 0.047 | 0.061 | 0.133 |
| *DA052* (*Acidobacteria*) | 0 | 0.564 | 0.003 |
| *Thermoleophilia* (*Actinobacteria*) | 0.248 | 0.619 | 0.043 |
| *Acidobacteriia* (*Acidobacteria*) | 0.001 | 0.206 | 0.852 |
| *Nitrospira* (*Nitrospirae*) | 0.895 | 0 | 0.347 |
| *Saprospirae* (*Bacteroidetes*) | 0.057 | 0 | 0.616 |
| *Actinobacteria* (*Actinobacteria*) | 0.334 | 0 | 0.309 |
| *iii1_8* (*Acidobacteria*) | 0.805 | 0 | 0.013 |
| *Ellin6529* (*Chloroflexi*) | 0 | 0.497 | 0.222 |
| *ABS_6* (*AD3*) | 0 | 0 | 0.015 |
| *Planctomycetia* (*Planctomycetes*) | 0 | 0 | 0 |
| *Solibacteres* (*Acidobacteria*) | 0.094 | 0.057 | 0.049 |
| *Acidobacteria_6* (*Acidobacteria*) | 0.465 | 0.093 | 0.677 |
| *Gemm_1* (*Gemmatimonadetes*) | 0.200 | 0 | 0.022 |

**Table S6** Partial spearman’s rank correlations between soil bacterial richness and diversity (Shannon’s diversity, phylogenetic diversity) and environmental parameters (5 variables which have significant relationships with bacterial diversity according to correlation analysis) in deep layer.

|  | Observed OTUs | Shannon’s diversity | Faith's phylogenetic diversity |
| --- | --- | --- | --- |
| MAP | 0.402 | 0.371 | 0.545 |
| Shannon index of trees | 0.159 | 0.095 | -0.082 |
| M_o_:SOC | 0.097 | 0.458 | 0.212 |
| LPII% | **0.709** | **0.557** | 0.440 |
| pH | **0.559** | **0.647** | 0.440 |

Numbers in bold indicate significant correlations with *p*<0.05.

**Table S7** The rRNA operon copy number for the main taxa at genus level according to the rrnDB database.

| genus level | Range | Medium | Mean |
| --- | --- | --- | --- |
| Candidatus Koribacter (*Acidobacteria*) | 1-1 | 1 | 1 |
| Candidatus Solibacter (*Acidobacteria*) | 2-2 | 2 | 2 |
| g_; Syntrophobacteraceae (*Delta-proteobacteria*) | 2-2 | 2 | 2 |
| Geobacter (*Delta-proteobacteria*) | 2-4 | 2 | 2.7 |
| g_; Myxococcaceae (*Delta-proteobacteria*) | 3-4 | 4 | 3.7 |
| Gemmatimonas (*Gemmatimonadetes*) | 1-2 | 2 | 1.7 |
| g_; Pirellulaceae (*Planctomycetes*) | 1-2 | 1 | 1.2 |
| g_; Gemmataceae (*Planctomycetes*) | 2-5 | 3 | 3.6 |
| g_; f_; o_H39; c_Anaerolineae (*Chloroflexi*) | 1-2 | - | 1.5 |
| Nitrospira (*Nitrospirae*) | 1-3 | 1 | 1.6 |
| Bradyrhizobium (*Alpha-proteobacteria*) | 1-2 | 1 | 1.1 |
| Rhodoplanes (*Alpha-proteobacteria*) | 1-1 | 1 | 1 |
| Mesorhizobium (*Alpha-proteobacteria*) | 1-3 | 2 | 1.9 |
| Arthrobacter (*Actinobacteria*) | 2-13 | 5 | 5.4 |
| g_; Thermomonosporaceae (*Actinobacteria*) | 4-6 | 5 | 4.8 |
| Flavobacterium (*Bacteroidetes*) | 1-13 | 6 | 7.2 |
| g_; Chitinophagaceae (*Bacteroidetes*) | 1-7 | 2 | 3 |
| Niabella (*Bacteroidetes*) | 2-2 | 2 | 2 |
| Paenibacillus (*Firmicutes*) | 6-15 | 10 | 10.6 |
| Burkholderia (*Beta-proteobacteria*) | 1-10 | 3 | 3.6 |
| Janthinobacterium (*Beta-proteobacteria*) | 1-10 | 8 | 6.7 |
| Pseudomonas (*Gamma-proteobacteria*) | 1-9 | 5 | 4.9 |
| Acidithiobacillus (*Gamma-proteobacteria*) | 2-2 | 2 | 2 |

The rRNA operon copy number for each taxa was estimated through the rrnDB database (https://rrndb.umms.med.umich.edu/) (Stoddard et al., 2015). “g_;” means the taxa was not identified at the genus level; “f_;” means the taxa wasn’t identified at the family level. For the unidentified taxa at the genus level, the copy number of its parent is used.

Stoddard S.F, Smith B.J., Hein R., Roller B.R.K. and Schmidt T.M. (2015) rrnDB: improved tools for interpreting rRNA gene abundance in bacteria and archaea and a new foundation for future development. Nucleic Acids Research 2014; doi: 10.1093/nar/gku1201 [PMID:25414355]
